# Advances in CRISPR-Cas technology and its applications: revolutionising precision medicine

**DOI:** 10.3389/fgeed.2024.1509924

**Published:** 2024-12-12

**Authors:** Sarkar Sardar Azeez, Rahin Shareef Hamad, Bahra Kakamin Hamad, Mudhir Sabir Shekha, Peter Bergsten

**Affiliations:** ^1^ Department of Medical Laboratory Technology, Soran Technical College, Erbil Polytechnic University, Erbil, Kurdistan Region, Iraq; ^2^ Nursing Department, Soran Technical College, Erbil Polytechnic University, Erbil, Kurdistan Region, Iraq; ^3^ Department of Medical Laboratory Technology, Erbil Health and Medical Technical College, Erbil Polytechnic University, Erbil, Kurdistan Region, Iraq; ^4^ Department of Biology, College of Science, Salahaddin University, Erbil, Kurdistan Region, Iraq; ^5^ Department of Medical Cell Biology, Uppsala University, Uppsala, Sweden

**Keywords:** CRISPR-Cas9, genome editing, epigenome modulation, cancer immunotherapy, and CRISPR in clinical trials

## Abstract

CRISPR-Cas (Clustered Regularly Interspaced Short Palindromic Repeats-CRISPR-associated proteins) has undergone marked advancements since its discovery as an adaptive immune system in bacteria and archaea, emerged as a potent gene-editing tool after the successful engineering of its synthetic guide RNA (sgRNA) toward the targeting of specific DNA sequences with high accuracy. Besides its DNA editing ability, further-developed Cas variants can also edit the epigenome, rendering the CRISPR-Cas system a versatile tool for genome and epigenome manipulation and a pioneering force in precision medicine. This review explores the latest advancements in CRISPR-Cas technology and its therapeutic and biomedical applications, highlighting its transformative impact on precision medicine. Moreover, the current status of CRISPR therapeutics in clinical trials is discussed. Finally, we address the persisting challenges and prospects of CRISPR-Cas technology.

## Graphical Abstract

**Figure:**
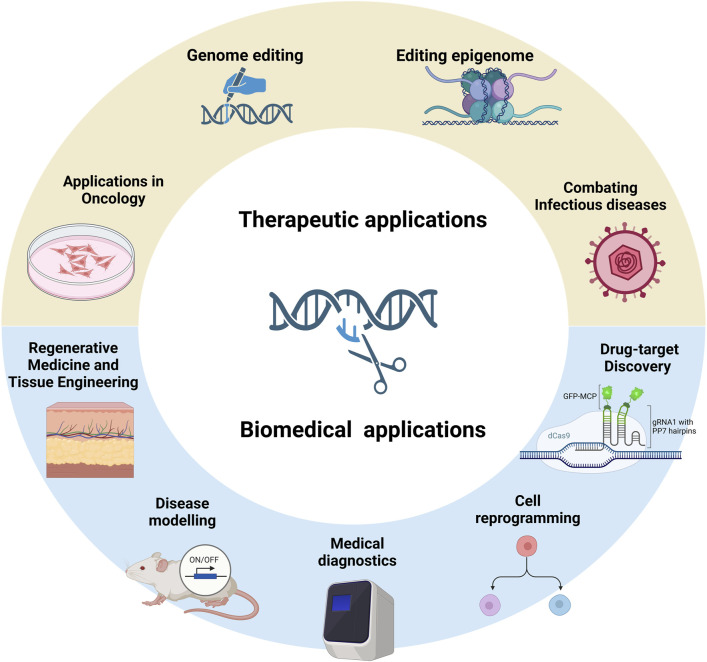

## 1 Introduction

CRISPR-Cas systems are an acquired immune system of most bacteria and archaea, protecting them from invading viruses, bacteriophages or mobile genetic elements (Jinek et al., 2012). The critical components of the CRISPR-Cas system include CRISPR-associated (Cas) proteins and the CRISPR array (Huang et al., 2018). Cas9, a well-characterized protein in this system, features endonuclease domains (HNH and RuvC) and a gRNA-binding domain (REC) (Wang et al., 2022a). The CRISPR array consists of short repetitive DNA sequences separated by spacers derived from prior infections (Doudna and Charpentier, 2014).

Upon re-infection, the array is transcribed into precursor CRISPR RNA (pre-crRNA) and processed into mature crRNAs, which, along with trans-activating CRISPR RNA (tracrRNA), guide Cas proteins to the target DNA. A protospacer adjacent motif (PAM) site adjacent to the target is recognised for binding, enabling Cas proteins to introduce double-stranded breaks (DSBs) at the target site and inactivating the virus (Jore et al., 2012; Barrangou and Marraffini, 2014).

The mechanistic discovery of CRISPR-Cas’s principle of action over decades of research culminated in 2012 in the engineering of crRNAs into a single, synthetic guide RNA designed to target specific sequences, with the potential of developing as a versatile genome editing tool (Gasiunas et al., 2012; Jinek et al., 2012). Shortly after, it was demonstrated that CRISPR-Cas9 technology enables effective and targeted genome editing in mammalian cells when they showed that DSBs created by Cas9 trigger cellular DNA repair pathways such as non-homologous end joining (NHEJ) or homology-directed repair (HDR). NHEJ often results in insertions or deletions (indels), causing gene disruptions (Silva et al., 2019). HDR, on the other hand, is a high-fidelity DNA repair pathway that can be used with a donor template to induce particular genetic alterations (Liao et al., 2024).

This groundbreaking innovation has enabled accurate and specified genomic modifications more easily adapted than traditional gene editing techniques, such as zinc-finger nucleases (ZFNs) and transcription activator-like effector nucleases (TALENs) (Gaj et al., 2013). While ZFNs and TALENs can also facilitate targeted genomic editing, but they are limited by their complex and labour-intensive design and assembly processes, which are less practical for rapid and versatile applications. In contrast, CRISPR technology offers a simpler, more efficient, and highly adaptable system. Its guide RNA-based targeting mechanism allows quick design and broad applicability across different genomic targets (Gupta and Musunuru, 2014). This feature has given CRISPR-Cas technology tremendous adaptability and utility in numerous biological areas such as genetic engineering, functional genomics, and medicinal development.

The present study comprehensively reviews the latest advances in CRISPR-Cas technology and its applications, underscoring its revolutionary impact on precision medicine. Its medical applications have been divided into therapeutic and biomedical. The therapeutic use of CRISPR-Cas-based genome and epigenome editing includes correcting genetic disorders, antiviral therapy, and eliminating antimicrobial resistance. It has been widely applied in oncology due to its efficiency in engineering chimeric antigen receptor T-cell (CAR-T cell) therapies and oncolytic viruses, targeting oncogenes, and modifying the tumour microenvironment. Biomedical applications include drug target discovery, modelling diseases, regenerative medicine, tissue engineering, cell reprogramming, and medical diagnostics. Finally, the current status of CRISPR therapeutics in clinical trials and the remaining challenges in the applications of CRISPR-Cas are discussed.

## 2 An overview of CRISPR-Cas system

### 2.1 CRISPR-Cas systems, classes, and types

**FIGURE 1:**
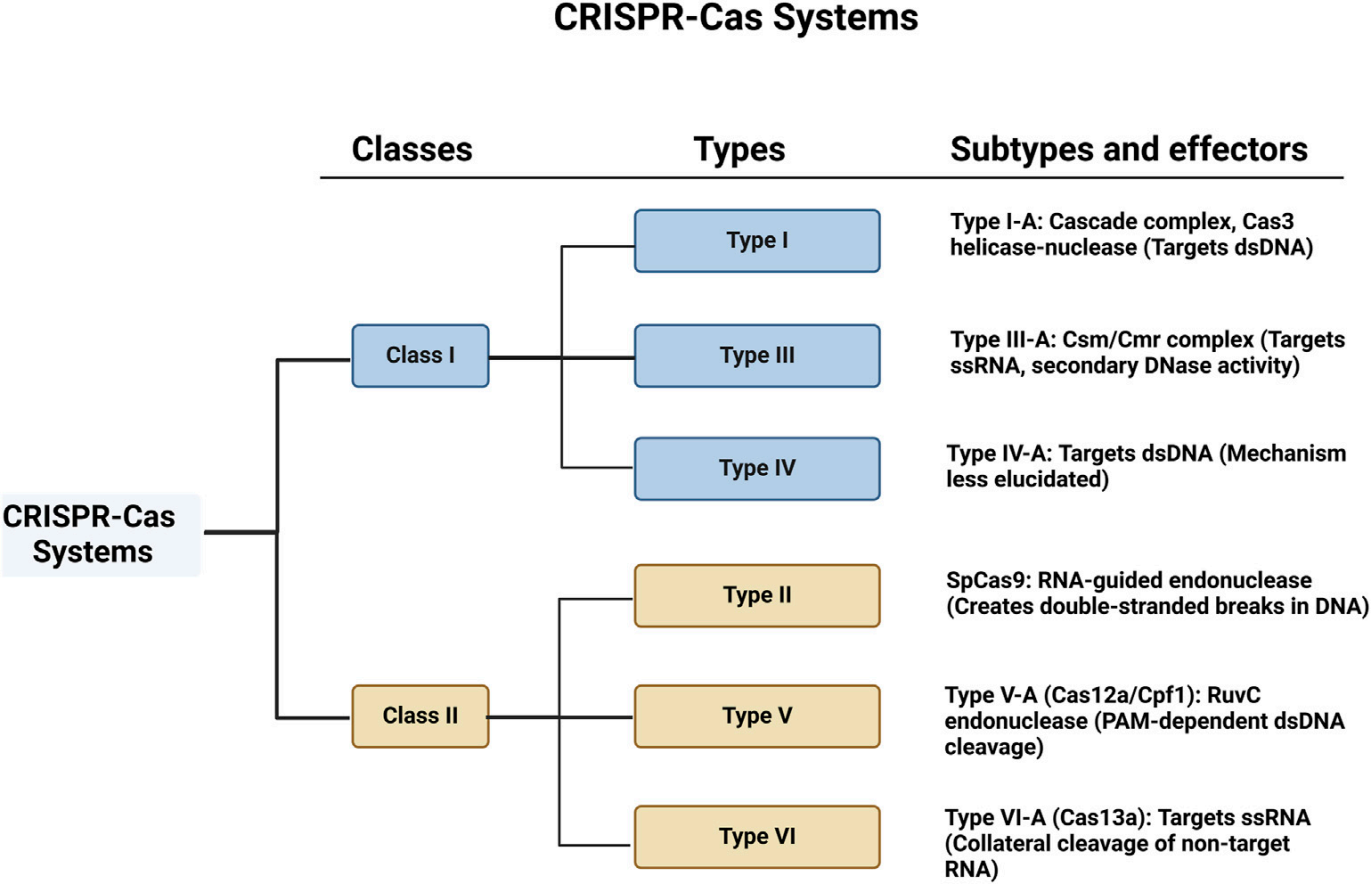
Schematic representation of CRISPR-Cas systems, categorised into Class 1 and Class 2. Each class is further divided into types and subtypes, showing the associated complexes and their primary targets.

CRISPR-Cas systems exhibit significant diversity across prokaryotic organisms, and they are categorised into two major classes and six types based on their components and mechanisms of action (Figure 1). Class 1 systems, encompassing Types I, III, and IV, feature multi-protein effector complexes, while Class 2 systems, comprising Types II, V, and VI, are defined by single-protein effectors (Mohanraju et al., 2016).

Class 1 systems are more complex, with multiple proteins contributing to their function. Among these, Type I systems, such as Type I-A, utilise a CRISPR-associated complex for antiviral defence, Cascade (CRISPR-associated complex for antiviral defence), to recognise and bind complementary sequences in dsDNA. The Cas3 helicase-nuclease is recruited upon target recognition to unwind and directionally degrade the DNA. This system has been studied for its role in bacterial immunity but is less developed for genome editing due to its complexity (Yoshimi and Mashimo, 2022).

Type III systems, such as Type III-A, use the Csm/Cmr complex to target single-stranded RNA (ssRNA) while also engaging in a secondary DNase activity that targets nearby dsDNA, adding a layer of defence. These systems can cleave RNA and DNA, with applications in bacterial immunity and potentially in antiviral therapeutic approaches. However, the dual cleavage activity complicates its use in precision genome editing (Paraan et al., 2023). Type IV systems, like Type IV-A, also target dsDNA, but their detailed mechanisms remain less elucidated (Xu and Li, 2020).

Class 2 systems, which are simpler due to their single-protein effectors, have been the focus of most genome editing research. Class 2 systems include the well-studied Type II, with SpCas9 from *Streptococcus* pyogenes being a prominent member. SpCas9 has become the archetype for genome editing, with its precise DNA recognition and cleavage guided by sgRNA, targeting sequences adjacent to PAM, typically NGG, which is necessary for Cas9 binding and activation (Marraffini, 2016).

Type V systems, such as Type V-A (Cas12a/Cpf1), employ a single RuvC endonuclease domain to cleave target dsDNA in a PAM-dependent manner, followed by non-target strand cleavage (Liao et al., 2018; Paul and Montoya, 2020). Cas12a requires a T-rich PAM (e.g., TTTV), broadening the range of editable sequences compared to SpCas9’s G-rich PAM. Cas12a also performs staggered cuts in DNA, generating “sticky ends” that are advantageous for certain genetic modifications. Moreover, Cas12a can process its crRNA array independently, enabling more efficient multiplexed genome editing without requiring additional tracrRNA sequences (Paul and Montoya, 2020).

Lastly, type VI systems, represented by Cas13a, are distinct in their ability to target and cleave ssRNA rather than DNA (Watanabe et al., 2019). Upon binding to its target RNA, Cas13a exhibits a unique collateral cleavage activity, indiscriminately cutting nearby non-target RNAs (Liu L. et al., 2017). The structural and functional classifications of CRISPR-Cas systems are elaborated in greater detail in (Makarova and Koonin, 2015). The CRISPR-Cas systems continue to be refined for both basic research and clinical use, with each type offering unique properties that can be tailored to specific applications.

### 2.2 Technological advances in CRISPR-Cas nucleases and editing strategies

#### 2.2.1 Genome editing tools

**FIGURE 2:**
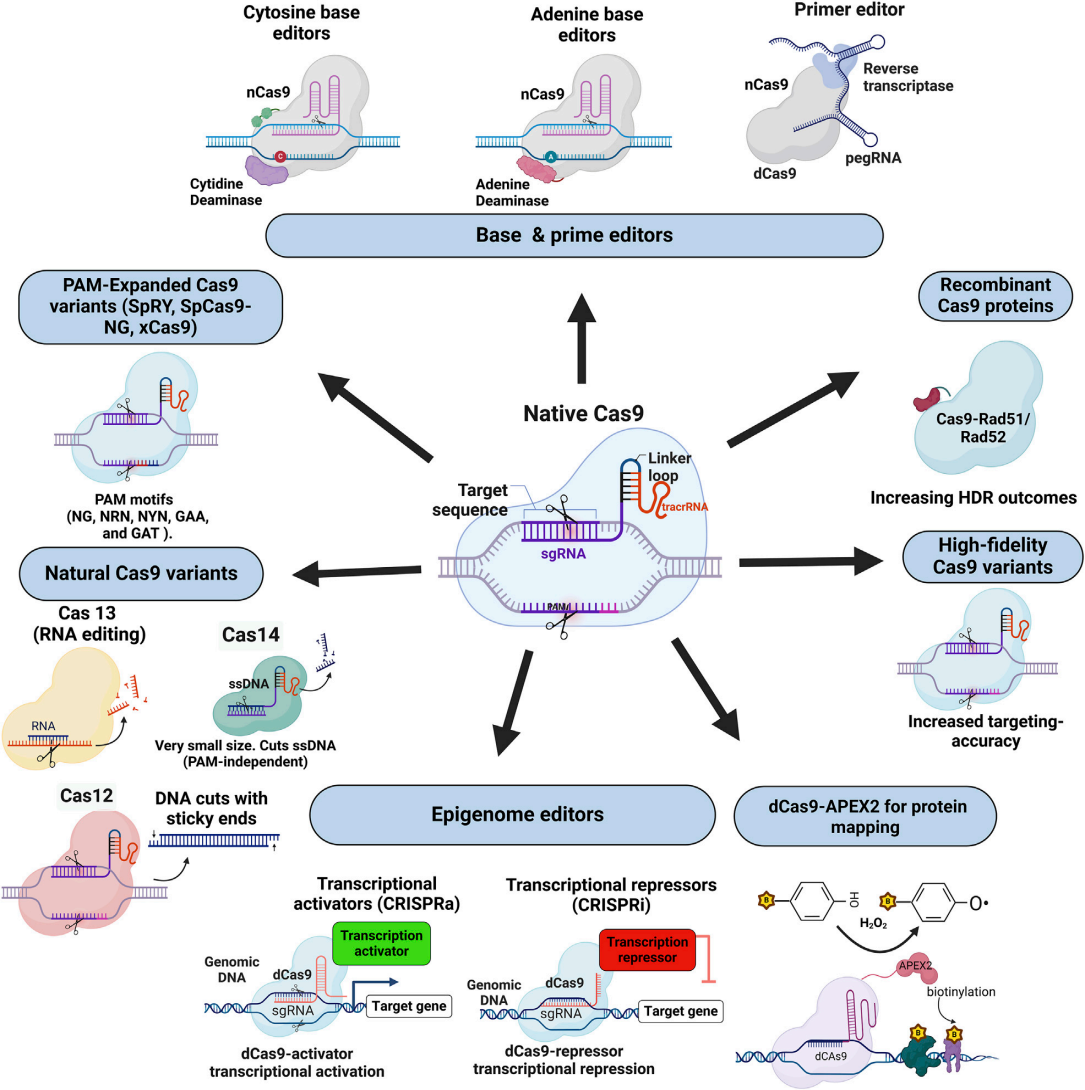
This figure displays a range of Cas protein variants and engineered tools derived from the CRISPR-Cas family, highlighting their diverse applications in genome and transcriptome editing. Central to the diagram is the native Cas9-sgRNA complex, which serves as a foundation for multiple modifications. Shown clockwise from the top left, base and prime editors (e.g., cytosine and adenine base editors, prime editors) allow single-nucleotide changes and precise insertions without DSBs. Recombinant Cas9 proteins (Cas9-Rad51/Rad52 fusions) enhance homology-directed repair (HDR) outcomes, while high-fidelity Cas9 variants reduce off-target effects to improve targeting specificity. Transcriptional activators (CRISPRa) and repressors (CRISPRi) enable gene expression modulation without altering DNA sequences, using dCas9 fused to transcriptional regulators. The dCAS9-APEX2 complex, tags nearby proteins with biotin, which is useful in proteome mapping. PAM-expanded Cas9 variants (e.g., SpRY, SpCas9-NG) target a broader range of PAM sequences, increasing flexibility in target selection. In the lower-left corner, “Natural Cas9 Variants” encompass Cas12, which produces sticky-end DNA cuts; Cas13, an RNA editor targeting RNA without modifying DNA; and Cas14, a small-size protein that cuts single-stranded DNA, independent of PAM recognition. This diverse toolkit illustrates the flexibility and breadth of CRISPR-Cas systems for targeted genetic and epigenetic modifications across various applications.

Over the past 30 years, CRISPR-Cas technology has evolved dramatically, significantly advancing the field of genetic engineering and emphasising its versatility and precision (Figure 2). The foundational Cas9 nuclease marked a turning point by enabling targeted DSBs in DNA, which facilitated precise gene editing (Jinek et al., 2012). Due to the simplicity of its action and design, Cas9 has been recognised as a powerful tool in research and clinical applications.

The traditional CRISPR-Cas9 editing relies on inducing DSBs at target sites. These DSBs can be repaired through the error-prone NHEJ pathway, probably leading to indels, or through HDR, which can be exploited to precisely incorporate donor DNA templates, rendering it a potent gene-editing tool in the correction of genetic disorders (Xue and Greene, 2021). However, the native Cas9 has several limitations despite its utility, such as off-target effects and strict dependence on NGG PAM sequences, which can restrict editing in certain genomic regions (Zhang et al., 2015; Sato and Kuroda, 2023).

The research innovations have focused on improving the specificity and efficiency of CRISPR-Cas systems. Some modified versions of Cas9, like high-fidelity Cas9 (SpCas9-HF1) and enhanced specificity Cas9 (eSpCas9), have been developed with decreased off-target effects by modulating protein-DNA interaction dynamics which minimise unintended edits in non-target genomic regions (Huang et al., 2022b). Studies have discovered that these high-fidelity Cas9 mutants achieve improved specificity due to a proofreading mechanism that keeps them inactive when encountering mismatched DNA sequences. This mechanism prevents unintended cuts, enhancing the precision of genome editing (Mengstie et al., 2024).

Another effective approach to minimise off-target effects is using CRISPR nickases, which modify one nuclease domain to cut only a single DNA strand. Unlike standard Cas9, which creates DSBs, Cas9 nickase introduces single-stranded “nicks” that cells can promptly repair, reducing collateral damage and off-target mutations (Trevino and Zhang, 2014). Paired nickases—targeting both DNA strands but separately, enhance precision by creating DSB-like edits with minimised off-target effects, making them highly advantageous for accurate genome editing (Torella et al., 2024, p. 1).

Furthermore, expanding the target sequence recognition depending on different PAM sequences has broadened its applications. The traditional Cas9 nucleases entirely depend on the PAM sequence—specifically, the NGG. This dependence on a specific PAM motif can be a significant limitation, as it restricts the range of possible target sites across the genome, particularly in genomic regions where the NGG motif is sparse or absent. This limitation has spurred the development of PAM-less or PAM-relaxed Cas9 variants, with SpRY as one of the most advanced examples. Engineered through mutations in its PAM-interacting domain, SpRY can recognise a broader range of PAM sequences, including NRN and NYN (where “R” is a purine and “Y” is a pyrimidine) (Hibshman et al., 2024). This flexibility allows SpRY to target nearly any genomic site, greatly expanding editing possibilities, particularly in AT-rich regions where NGG motifs are sparse (Walton et al., 2020). In addition to SpRY, other Cas9 variants with expanded PAM compatibility have been developed, including SpCas9-NG, which targets NG PAMs, and xCas9, which also recognises NG, GAA, and GAT PAMs (Hu et al., 2018; Hua et al., 2019). While these variants improve targeting flexibility, SpRY represents a more substantial breakthrough due to its ability to eliminate the PAM constraint effectively. This advancement has significant implications for therapeutic genome editing, as it provides greater flexibility to target disease-causing mutations regardless of PAM availability (Liang et al., 2022). Variants like SpCas9-HF1 and eSpCas9 minimise off-target edits, and SpRY and SpCas9-NG enable expanded PAM Compatibility. However, while improving specificity, some high-fidelity Cas9 variants might suffer from reduced cutting efficiency (Moreb et al., 2020; Kulcsár et al., 2022). Further optimisation is required to balance specificity and activity. Strategies such as fine-tuning protein-DNA interaction dynamics or enhancing guide RNA design could help improve cleavage efficiency while maintaining high specificity (Ryan et al., 2017).

Base editors enable targeted single-base pair conversions, thereby reducing the off-target effects and improving the accuracy of genome editing, hence very useful for correcting genetic disorders caused by point mutations (Komor et al., 2016; Liang et al., 2023). Base editors enable the accurate correction of point mutations and eliminate the need for donor templates. However, potential unintended base conversions can still occur at nearby bases and can only change one base at a time, limiting their application to large-scale edits (Doman et al., 2020; Jeong et al., 2020).

Prime editors enable several genome editing options, including base substitutions, insertions, and deletions of longer DNA sequences. Prime editors utilise a Cas9 nickase (nCas9) fused with a reverse transcriptase and a prime editing guide RNA (pegRNA), offering greater versatility for a broader range of genetic modifications (Anzalone et al., 2019). These editors represent a significant advancement in Cas9 engineering and a wide array of gene editing applications, including base substitutions, insertions, and deletions with precision. Also, the risk of off-target mutagenesis and safety for therapeutic applications is much reduced compared to other editing systems (Anzalone et al., 2019; Chen and Liu, 2023). However, reduced efficiency, the need for optimisation for different cell types and the complex design of pegRNA and its components make it more technically demanding (Zhao et al., 2023).

Further advancements include developing alternative CRISPR systems, such as Cas12 and Cas13. Cas12, which makes staggered cuts in DNA, is particularly useful for insertions and genetic modifications that require sticky ends. Its smaller size and distinct PAM requirements make it a versatile tool for applications where Cas9’s blunt cuts and NGG PAM are limiting (Zetsche et al., 2015; Chen et al., 2018).

Cas14 is an ultra-small CRISPR-associated protein discovered in certain archaea, notable for its size, typically under 70 kDa, compared to the larger Cas9 and Cas12 proteins (Savage, 2019). This smaller size makes Cas14 advantageous for gene-editing applications requiring compact delivery systems, like adeno-associated viruses (AAVs), which have limited payload capacity (Harrington et al., 2018). Cas14 uniquely targets single-stranded DNA (ssDNA) via target binding with the gRNA without the need for a protospacer adjacent motif (PAM), broadening its potential target range and making it highly versatile for applications in diagnostics, gene therapy, and microbial studies (Harrington et al., 2018).

On the other hand, identifying RNA targeting Cas13 opened a new avenue for RNA editing. Indeed, the RNA editing potential of Cas13 has been exploited to detect viral RNA, including SARS-CoV-2, using its collateral cleavage activity targeting viral RNA sequences (Abudayyeh et al., 2017; Zhou et al., 2022). This mechanism has been leveraged for molecular diagnostics, such as the detection of viral RNA, and holds promise in developing treatments for RNA viruses (Yin et al., 2020). Cas13a′s specificity for RNA without DNA modification makes it an attractive tool for transient knockdowns in research and potential therapeutic applications for diseases involving RNA, such as certain viral infections and neurodegenerative disorders (Makarova and Koonin, 2015). While Cas13’s RNA specificity is ideal for transient knockdowns and diagnostics, its collateral cleavage can sometimes result in off-target RNA degradation, limiting its therapeutic potential (Ai et al., 2022; Li et al., 2023). These improvements in the functioning Cas enzymes underlie the transformative impact and advancement in applying CRISPR-Cas technology in precision medicine and treatment.

#### 2.2.2 Genome editing strategies

Gene editing relies on precise, programmable nucleases to generate specific genetic alterations. Key editing strategies include knockouts, gene insertions, and epigenetic modifications. The CRISPR-Cas9 system facilitates gene knockouts primarily by introducing indels at specific target sites (Canver et al., 2014). Indels are generated when the Cas9 nuclease induces a DSB in DNA at a specific site, prompting the NHEJ repair pathway (Jinek et al., 2012; Xue and Greene, 2021). Due to the error-prone nature of NHEJ, insertions or deletions are randomly introduced at the cleavage site, often resulting in frameshift mutations (Rodgers and McVey, 2016). This mutation disrupts the open reading frame of the targeted gene, typically resulting in a non-functional protein and an effective gene knockout (Tsutsui and Higashiyama, 2016). This indel-based strategy has proven effective in knocking out single genes, with applications spanning from basic research to therapeutic interventions, such as eliminating defective genes in monogenic diseases (Guo et al., 2018).

For larger deletions, dual Cas9 nucleases, each targeted to different loci flanking a region of interest, enable the excision of extensive DNA sequences. By employing two Cas9 nucleases simultaneously, each guided to distinct loci flanking the target gene region, a large segment of DNA can be excised between the two DSBs (Zhou et al., 2014; Cai et al., 2018). This approach effectively deletes entire gene regions, introns, or regulatory sequences distant from the coding region. The resulting deletions can span several kilobases, allowing for the removal of larger, structurally complex genes or multiple adjacent genes. Dual Cas9-mediated deletions are particularly valuable in studying genetic redundancies and the function of large genes or gene clusters within complex genomic regions (Adikusuma et al., 2018; Eleveld et al., 2021).

In contrast, gene knock-ins, which require the precise insertion of genetic material, are often mediated through homology-directed repair (HDR). Following Cas9-induced DSBs, HDR uses a donor DNA template to introduce new genetic material into the break site, enabling precise alterations such as replacing a defective gene with a functional allele (Yao et al., 2018).

Recently, researchers fused Cas9 with recombinases, such as Rad51 or RAD52, which enhance homology-directed repair (HDR) by promoting alignment and integration of donor DNA templates at Cas9-induced DSBs (Tran et al., 2019). These fusions increase HDR efficiency by directing repair machinery toward precise edits while reducing indel formation typical of NHEJ. Such strategies improve editing precision, which is especially valuable in therapeutic contexts requiring exact gene corrections (Shao et al., 2017).

However, HDR is most active during the S and G2 phases of the cell cycle, which limits its application in non-dividing cells (Rein et al., 2018). Alternative pathways, such as microhomology-mediated end joining (MMEJ), have been explored for gene insertion in non-dividing cells, although their efficiency and fidelity vary (Nakade et al., 2014). Precise gene knock-ins are pivotal in functional genomics, disease modelling, and therapy development. It enables precise insertion of DNA sequences, facilitating gene function and protein localisation studies and creating disease models by introducing specific mutations. In therapeutics, CRISPR knock-ins allow the correction of genetic mutations, offering potential treatments for genetic disorders (Xu and Li, 2020).

Beyond direct gene changes, Cas9-mediated indels disrupting the promoter and enhancer region can also modulate gene expression. This strategy involves targeting indels specifically within the promoter and enhancer sequences to impair binding sites for transcription factors, thus reducing or silencing gene expression (Canver et al., 2015). This strategy enables researchers to modulate gene expression without directly altering coding sequences, which is particularly valuable in studying gene regulatory elements and developing therapeutics for overexpressed oncogenes.

#### 2.2.3 Epigenome editing tools and editing strategies

Beyond gene editing, CRISPR-Cas can control gene expression without altering the DNA sequence through epigenetic editing using catalytically inactive or dead Cas9 (dCas9) fused to epigenetic effectors (Figure 2). A primary objective of epigenetic editing includes alterations in DNA methylation patterns, a key epigenetic modification that typically results in the suppression of gene expression. This is achieved by combining the dCas9 with DNA methyltransferases such as DNMT3A to add methyl groups to specific CpG sites (Xiong et al., 2017). On the other hand, CRISPR-based DNA demethylation is obtained by combining dCas9 with DNA demethylases, such as TET1. This approach enables the selective removal of methyl groups in specific genomic regions (Morita et al., 2016). This strategy has been effectively utilised to manipulate gene expression patterns, providing a method to reverse the impact of epigenetic silencing in certain diseases where hypermethylation is a contributing factor.

Histone modifications, including acetylation, methylation, and phosphorylation, are another key focus of CRISPR-Cas-mediated epigenome editing. dCas9 can be coupled with histone acetyltransferases (HATs) like p300 or histone deacetylases (HDACs) to add or remove acetyl groups on histones. These modifications can activate or repress gene expression by altering chromatin accessibility (Hilton et al., 2015; Kwon et al., 2017). For example, dCas9-p300, a histone acetyltransferase, has been used to increase histone acetylation at the promoter regions of IL1RN, MYOD, and OCT4 genes, leading to a significant transcriptional activation of the corresponding genes (Hilton et al., 2015). Conversely, dCas9-HDACs have decreased acetylation at promoter regions, thereby repressing their expression (Kwon et al., 2017).

Chromatin remodelling is another critical aspect of gene regulation that can be modulated using CRISPR-Cas technology. By fusing dCas9 to chromatin remodelling complexes, researchers can reposition nucleosomes and alter chromatin structure at specific genomic loci (Ding et al., 2019). This capability is vital for regulating otherwise inaccessible genes due to tightly packed chromatin. Targeted chromatin remodelling has the potential to reactivate silenced genes, offering new therapeutic avenues for diseases where gene repression is mediated by chromatin compaction (Li et al., 2021b).

Moreover, CRISPR-Cas can directly promote gene expression through CRISPR activation (CRISPRa) systems. CRISPRa involves using dCas9 that is fused to transcriptional activators, such as the VP64 domain, p65, or the Rta (Epstein-Barr virus transcriptional activator) (Omachi and Miner, 2022). When directed to a specific promoter region by a gRNA, the dCas9-activator complex enhances the transcription of the target gene (Casas-Mollano et al., 2020). This mechanism allows for the upregulation of gene expression, effectively mimicking the natural activity of endogenous transcription factors. Conversely, CRISPR Interference (CRISPRi) involves the combination of dCas9 to transcriptional repressors, such as the KRAB (Kruppel-associated box) domain, to inhibit gene expression (Li et al., 2021a). When the dCas9-KRAB complex is directed to a promoter or enhancer region, it recruits additional co-repressors and chromatin remodelers, leading to the formation of a repressive chromatin environment that silences gene expression (Yeo et al., 2018). This method is beneficial for studying gene function and therapeutic applications where gene silencing is desired.

Epigenetic editing strategies through CRISPR-Cas systems have been further expanded to modulate non-coding RNAs (ncRNAs) and their regulatory functions within the genome. CRISPR technology alters non-coding RNAs (ncRNAs) through several innovative approaches. One method involves direct editing of ncRNA sequences using CRISPR-Cas13. This RNA-targeting enzyme allows for direct degradation and reduction in the levels of targeted ncRNAs (Abudayyeh et al., 2017; Hazan and Bester, 2021). Additionally, CRISPR-Cas can be employed to modulate ncRNA expression indirectly. For example, CRISPRi can inhibit ncRNA gene expression, while CRISPRa enhances ncRNA expression (Liu et al., 2017c). These methods enable researchers to study and manipulate ncRNAs to understand their involvement in gene regulation and various biological processes.

Finally, the dCas9 is fused to APEX2, an enzyme that labels nearby proteins with biotin, enabling targeted proteomic mapping (Gao et al., 2018). This protein labelling system enables precise biotinylation and high-throughput identification of proteins associated with selected genomic regions, shedding light on chromatin’s architecture and gene expression regulation (Dolgalev and Poverennaya, 2021).

The epigenetic modifications offer a reversible and controlled approach to gene regulation, potentially useful in reprogramming cells, studying gene regulatory networks, and developing therapeutic strategies for diseases linked to epigenetic dysregulation. However, the off-target effects and cytotoxicity are some of the major concerns using these types of editors (Cai et al., 2023). Also, they are of limited duration and might require continuous expression of the dCas9 system, and the effectiveness of epigenetic editing can vary based on chromatin accessibility and other epigenetic factors (Whittaker et al., 2023).

#### 2.2.4 gRNA modifications

gRNAs are essential for the functionality of CRISPR-Cas systems, as they enable precise targeting by guiding Cas9 to specific genomic loci based on sequence complementarity (Asmamaw and Zawdie, 2021). In recent years, substantial efforts have been directed towards engineering synthetic gRNAs to enhance the stability, specificity, and efficacy of CRISPR-Cas9, particularly in therapeutic applications in mammalian cells.

Specific chemical modifications, such as adding 2′-O-methyl and phosphorothioate groups (MS), stabilise gRNA ends, protecting against exonuclease degradation and allowing for sustained activity within the cell (Basila et al., 2017). This stability has been crucial for extending gRNA lifespans in environments rich in nucleases, thereby enhancing editing efficiency without continuously replenishing gRNA (Basila et al., 2017). In a study, modified gRNA with MS or 2′-O-methyl 3′-thio PACE (MSP) bound with Cas9 protein electrotransferred into human primary T cells and CD34 + hematopoietic stem cells demonstrated a significant increase (2.4-fold) in indel formation compared to non-modified gRNAs (Hendel et al., 2015).

Additionally, internal modifications to gRNA, specifically in regions like the seed sequence (proximal to the protospacer adjacent motif or PAM site), have been explored to increase target specificity. Locked nucleic acids (LNAs) and bridged nucleic acids (BNAs) incorporated into the guide sequence reduce off-target effects by reinforcing RNA-DNA hybridisation in cases of precise base pairing while reducing binding affinity in cases of mismatch (Cromwell et al., 2018; Sakovina et al., 2022).

Moreover, reducing immune responses in mammalian cells is essential for therapeutic applications. By removing immunogenic elements, such as the 5′-triphosphate group that commonly activates intracellular immune pathways, and by introducing 2′-O-methyl groups, researchers have minimised inflammatory responses, rendering gRNAs more compatible with clinical use in primary cells (Wienert et al., 2018). Other advanced gRNA modifications also aim to augment homology-directed repair (HDR), critical for gene correction applications, by linking gRNA with donor DNA sequences to enhance proximity-based efficiency, thereby increasing the fidelity and control of HDR pathways in cellular contexts (Lee et al., 2017b). gRNA modifications significantly refine CRISPR-Cas9 efficacy, specificity, and safety by mitigating degradation, off-target effects, and immune reactions. This engineered precision enables safer gene therapies and supports functional genomics, cellular imaging, and disease modelling applications.

### 2.3 Anti-CRISPR proteins

Anti-CRISPR proteins (Acr) are naturally occurring antagonists of the CRISPR-Cas immune system, primarily discovered in bacteriophages, which evolved these proteins to counteract CRISPR-mediated immunity in bacteria (Marino et al., 2020; Gebhardt and Niopek, 2024). Mechanistically, Acr proteins inhibit CRISPR-Cas activity by interacting directly with Cas proteins to block DNA targeting or cleavage at various stages. For example, AcrIIA4 binds Cas9, obstructing its DNA-binding ability, while AcrVA1 enzymatically cleaves guide RNA in the Cas12a complex, preventing target recognition and cleavage (Kim et al., 2018; Knott et al., 2019). Acr proteins have critical applications, particularly in refining gene-editing techniques by providing controlled, post-translational inhibition of CRISPR-Cas systems. This targeted deactivation is particularly beneficial in therapeutic contexts, where high precision is crucial to avoid unintended gene alterations and cytotoxicity (Kraus and Sontheimer, 2023). Furthermore, Acr proteins can prevent CRISPR-Cas systems from targeting specific tissues, adding a layer of safety for CRISPR-based therapies. Additionally, they enable new strategies in phage therapy by enhancing the specificity of engineered bacteriophages against CRISPR-equipped bacterial pathogens (Qin et al., 2022).

## 3 Delivery strategies of CRISPR-Cas system

Gene therapy involves the efficient delivery of nucleic acids to repair mutations, add new cell functions, or modulate gene expression. However, delivery has limitations due to hydrolysis, low cellular uptake, and a negative surface charge. To overcome these issues, delivery vectors are employed and categorised into viral and non-viral systems (Abd Ellah et al., 2021). Viral vectors use modified viruses such as adenoviruses, retroviruses, and lentiviruses, enabling efficient gene transfer and high gene expression levels. However, they pose risks like immunogenicity, toxicity, and complications in large-scale production. There is also potential for insertional mutagenesis, where viral DNA integrates into the host genome, which could disrupt normal gene function and lead to complications, including cancer. Non-viral gene delivery systems are increasingly explored due to their safer profiles but generally have lower transfection efficiencies. These systems include physical techniques such as electroporation, microinjection, hydrodynamic delivery, and chemical methods like liposomes, polymers, and inorganic nanoparticles. Despite lower efficiency, non-viral systems have shown potential for safe gene therapy applications (Cevher et al., 2012).

### 3.1 Viral vectors

Viral vectors are among the most established methods for gene delivery, leveraging viruses’ natural ability to infect cells and deliver genetic material. In CRISPR-Cas systems, viral vectors are commonly employed to transport Cas9 and sgRNA components into target cells. The surface proteins of viruses can overcome cellular barriers, allowing effective cargo deposition (i.e., DNA, mRNA, and other materials) (Mengstie and Misganaw, 2022).

In recent decades, four major types of recombinant viral vectors have been used in gene therapy: adenovirus (AV), adeno-associated virus (AAV), lentivirus (LV), and gamma retrovirus (γ-RV). Adenoviruses (AVs) are double-stranded DNA viruses known for their efficiency in transducing a wide range of cell types. Unlike AAVs, AVs do not integrate into the host genome, leading to transient expression of the transgene (Wang et al., 2024a). AVs are highly efficient in transducing both dividing and non-dividing cells, making them suitable for *in vitro*, *in vivo*, and *ex vivo* applications. AVs can carry larger genetic payloads (∼8 kb), enabling the delivery of the more commonly used SpCas9 system. Because AVs do not integrate into the host genome, they are associated with lower risks of insertional mutagenesis, a significant consideration in therapeutic safety. However, AVs are highly immunogenic, and their use in humans can elicit robust immune responses. This immunogenicity limits their use in repeat dosing and, in some cases, can lead to inflammation or tissue damage. Also, AVs provide transient expression of Cas9, which may be a drawback for therapies requiring long-term expression and regulation (Lee et al., 2017a).

AAVs, non-pathogenic and helper-dependent for replication, are popular in gene therapy for delivering CRISPR-Cas9 to both dividing and non-dividing cells due to their persistence and low immunogenicity, which reduces adverse immune responses. A key advantage of AAVs is their capacity to deliver HDR templates for gene knock-in approaches, enabling precise genome editing (Duddy et al., 2024). They also offer extended gene expression, which is crucial for sustained therapies and can be engineered for specific tissue targeting. However, AAVs are limited by a packaging capacity of ∼4.7 kb and face challenges with repeat dosing, as immune memory against the capsid can hinder re-administration (Wang et al., 2019a). Moreover, recent findings indicate that in the context of CRISPR-Cas9 editing, AAV fragments can integrate into the genome. This occurs potentially via vector capture at DSBs introduced by Cas9 (Hanlon et al., 2019). These unintended integration events raise safety concerns, particularly in therapeutic applications, as they may lead to off-target genomic alterations or instability.

LVs, a retrovirus subclass, are widely used in gene therapy due to their ability to integrate genetic material into the host genome, enabling stable, long-term gene expression. This stability makes LVs ideal for applications requiring continuous CRISPR-Cas9 activity over time. With a packaging capacity of around 9 kb, LVs can accommodate larger Cas9 proteins and multiple sgRNAs, allowing for complex gene-editing tasks. Furthermore, lentiviral vectors can be engineered to target specific cell types by modifying their envelope proteins, enhancing therapeutic specificity across diverse tissues. However, genome integration presents a risk of insertional mutagenesis, which could disrupt essential genes or activate oncogenes, raising potential carcinogenic concerns (Hacein-Bey-Abina et al., 2003). To address these limitations, Integration-Deficient Lentiviral Vectors (IDLVs) have been developed. IDLVs are engineered with mutations in their integrase gene, rendering them unable to integrate into the host genome. This design allows them to deliver CRISPR-Cas9 components transiently, reducing the risk of insertional mutagenesis while maintaining efficient transduction, rendering it particularly advantageous for applications requiring episomal expressions, such as transient Cas9 activity or the delivery of HDR templates (Ortinski et al., 2017; Cortijo-Gutiérrez et al., 2021). Although IDLVs show promise, they often exhibit reduced expression longevity compared to integrative LVs, which could limit their applications in therapies requiring long-term gene correction (Yew et al., 2022). Also, while generally less immunogenic than adenoviruses, LVs can still elicit immune responses, especially *in vivo* applications (Dong and Kantor, 2021).

Retroviruses have been utilised to deliver CRISPR-Cas9 components due to their ability to integrate genetic material into host genomes, enabling long-term expression of the CRISPR system in dividing cells. This is particularly useful in therapies targeting proliferating cells, such as stem or hematopoietic cells. However, retroviral vectors are limited by their inability to target non-dividing cells, low *in vivo* transfection efficiency, and potential risks of insertional mutagenesis, which could disrupt host genes and lead to oncogenesis (Caffery et al., 2019).

### 3.2 Physical non-viral delivery methods

Physical methods, including electroporation, microinjection, and hydrodynamic injection, facilitate the delivery of CRISPR-Cas9 components by physically penetrating cellular barriers. Due to their technical limitations in live animals or humans, these methods are primarily used in *ex vivo* or *in vitro* settings.

#### 3.2.1 Electroporation

Electroporation involves the application of an electric field to cells, creating temporary pores in the cell membrane through which CRISPR-Cas9 components can enter. This technique is particularly effective for *ex vivo* applications, where cells can be modified outside the body and reintroduced into the patient (Pi et al., 2024). Electroporation allows for rapid and efficient delivery of CRISPR components into various cell types, including primary and stem cells (Qin et al., 2015). This method can deliver all CRISPR formats (plasmid DNA, mRNA, or protein), allowing application flexibility. However, the electric pulses used in electroporation can damage cell membranes, resulting in cell death, particularly in sensitive cell types. Moreover, electroporation parameters must be optimised for each cell type to balance efficiency and viability, complicating the protocol and limiting scalability for certain applications (Liu et al., 2017a).

#### 3.2.2 Microinjection

Microinjection is a precision technique in which CRISPR components are injected directly into cells using a microneedle under microscopic guidance. This method is typically applied to single cells, such as zygotes or embryos, and allows for highly controlled delivery. Microinjection provides precise control over the delivery of CRISPR components, minimising off-target effects. Microinjection allows for the delivery of any form of CRISPR components (DNA, RNA, protein), regardless of their molecular size. However, this technique is labour-intensive and unsuitable for large-scale applications, limiting its use primarily to research and preclinical studies. It also requires specialised equipment and expertise, adding complexity and limiting widespread adoption (Huang et al., 2022a).

#### 3.2.3 Hydrodynamic injection

Hydrodynamic injection involves rapidly infusing a large volume of solution containing CRISPR components into the bloodstream, creating increased pressure that facilitates cellular uptake. This method is most commonly applied in liver-targeted gene editing in animal models. Hydrodynamic injection is highly effective for liver-targeted delivery, making it useful for gene-editing therapies targeting hepatic diseases (Lino et al., 2018; Niola et al., 2019). This technique does not require complex equipment, making it relatively straightforward in preclinical studies. However, the large volume and high-pressure injection can damage blood vessels, cause liver stress, and induce cardiac side effects, limiting its application to animal models. Additionally, this method is effective primarily in the liver and has shown limited efficacy in targeting other tissues, reducing its versatility (Sinclair et al., 2023).

### 3.3 Chemical non-viral delivery methods

Chemical methods employ various nanoparticles and polymers to encapsulate and deliver CRISPR components into target cells. Nano-carriers deliver bio-macromolecular therapeutic agents like DNAs and RNAs to target cells, overcoming their degradation and ineffectiveness at crossing the cell-membrane barrier. These systems enhance the stability of these agents and protect them from premature degradation and rapid clearance *in vivo*.

#### 3.3.1 Lipid nanoparticles

Lipid nanoparticles (LNPs) are spherical vesicles composed of lipid bilayers commonly used to encapsulate nucleic acids, such as mRNA or Cas9 protein. They can facilitate cellular entry via endocytosis and enhance the cytoplasmic release of their payload. LNPs are generally biocompatible and biodegradable, reducing potential toxicity. LNPs can be functionalised to improve targeting specificity to certain cell types, such as hepatocytes, enhancing therapeutic efficacy (Han et al., 2022). Despite the advantages, LNPs have some limitations. LNPs may become trapped in endosomes upon entry into cells, requiring optimisation to ensure cytoplasmic release of CRISPR components. Some LNP formulations are prone to degradation in the bloodstream, necessitating further modifications for stability and circulation time (Kazemian et al., 2022).

#### 3.3.2 Polymer-based nanoparticles

Polymer-based nanoparticles are synthetic carriers made from biodegradable polymers like polyethyleneimine (PEI) or poly (lactic-co-glycolic acid) (PLGA). They form complexes with CRISPR-Cas components, enabling cellular uptake and controlled release. Polymer nanoparticles allow modification to optimise biocompatibility, release rates, and targeting capabilities (Khan, 2024). Polymer carriers can be engineered for sustained release, which is beneficial for applications requiring prolonged exposure to CRISPR components (Duan et al., 2021). The limitations of polymers, like PEI, include cytotoxicity at higher concentrations, limiting their safe dosage and applications (Kafil and Omidi, 2011). Furthermore, the synthesis of polymer nanoparticles requires extensive optimisation, and the manufacturing process can be more complex than other carriers (Zielińska et al., 2020).

#### 3.3.3 Inorganic nanoparticles

Inorganic nanoparticles, including gold or silica-based particles, are CRISPR-Cas systems carriers. These particles can form stable complexes with CRISPR components, particularly useful in imaging and tracking applications. Inorganic nanoparticles, such as gold, have unique optical properties that facilitate real-time tracking *in vivo*, aiding in monitoring the delivery process. Inorganic carriers exhibit high stability and can be designed for controlled release and precise targeting. However, inorganic nanoparticles may accumulate in tissues, raising concerns about long-term toxicity and clearance. Also, unlike organic carriers, many inorganic nanoparticles are not readily biodegradable, complicating their clinical applications (Zhou et al., 2024).

### 3.4 Other emerging delivery systems

Virus-like particles (VLPs) are noninfectious viral shells mimicking viral structure but lacking genetic material, making them a safer alternative to traditional viral vectors for delivering CRISPR components. These particles offer the benefit of high transduction efficiency with lower immunogenicity due to their lack of viral genome, enabling them to evade immune responses that viral vectors often trigger and rapid clearance, which reduces the risk of prolonged immune activation. Additionally, VLPs can be engineered for enhanced targeting specificity, enabling them to deliver Cas9 RNP complexes efficiently to specific cells. This approach shows promise for precise genome editing applications across various cell types. However, some synthetic peptides used for VLPs do not fully replicate viral structural functions, and production challenges remain for large-scale use due to complex assembly and potential heterogeneity (Rostami et al., 2024).

Exosomes, membrane-bound vesicles of about 30–150 nm, have gained attention for their ability to naturally transport genetic material with minimal immune response due to their endogenous origin. These vesicles show high biocompatibility, long circulation, and the ability to cross barriers like the blood-brain barrier. Due to these properties, exosomes are cell-derived vesicles used to deliver CRISPR components for gene editing in various diseases, with engineered exosomes potentially enhancing target cell-specific delivery and reducing off-target effects. Challenges in using exosomes include low production yield and difficulties in isolation, though scalable production and synthetic exosomes are being developed to overcome these issues. However, production challenges include heterogeneity, low yield, complex isolation/purification processes, and limited natural targeting ability without additional modifications (Rostami et al., 2024).

Advancing CRISPR-Cas gene-editing therapies relies heavily on developing efficient and safe delivery systems that can navigate complex biological barriers and minimise risks. Among the various delivery platforms explored, AAVs and LNPs have emerged as the most promising for *in vivo* CRISPR applications. With their proven track record in gene therapy, AAVs offer sustained gene expression, low immunogenicity, and tissue-targeting flexibility, making them ideal for applications requiring long-term therapeutic effects in both dividing and non-dividing cells. However, they are limited by packaging constraints and potential immune memory responses, which must be managed in therapeutic contexts requiring repeat doses (Wang et al., 2024b). On the other hand, LNPs bring versatility to CRISPR delivery by supporting the transient expression of CRISPR components, particularly Cas9 mRNA, which reduces the risk of prolonged genome editing and off-target effects. Their biocompatibility and capacity for molecular customisation enable efficient liver and lung targeting, and continuous advances in LNP formulations enhance their stability and endosomal escape efficiency (Mohammadian Farsani et al., 2024). Thus, AAVs and LNPs represent a balanced combination of efficacy, safety, and targeted delivery potential for *in vivo* CRISPR-Cas systems.

For *in-vitro* applications, electroporation remains an excellent method despite the challenges, offering high efficiency and versatility in CRISPR-Cas9 delivery across various cell types. Electroporation can facilitate optimal delivery with careful optimisation, making it a valuable tool for *ex vivo* therapeutic strategies where modified cells are reintroduced into the patient (Laustsen and Bak, 2019). As research progresses, optimising these platforms and developing new approaches will be essential to achieving precise and safe genome editing, ultimately bringing CRISPR closer to clinical and therapeutic applications. A comprehensive summary of the purpose, advantages, and limitations of various delivery systems for CRISPR-Cas technology, critical to its efficacy as a therapeutic agent, is provided in Table 1.

**TABLE 1:** Purpose, advantages, and limitations of delivery systems of CRISPR-Cas technology.

Delivery system	Purpose	Advantages	Disadvantages
Adenovirus (AV)	Broad gene delivery with transient expression	Efficient for various cell types, no genome integration (lower insertional mutagenesis risk), large capacity (∼8 kb)	Highly immunogenic, transient expression, limiting repeated dosing
Adeno-associated Virus (AAV)	Long-term gene expression in dividing/non-dividing cells	Low immunogenicity, long-term expression, tissue-targeting potential, and able to deliver HDR templates for gene knock-in experiments	Limited capacity (∼4.7 kb), risk of genome integration, and immune memory hinders re-administration
Lentivirus (LV)	Stable gene delivery by genome integration	Long-term expression, high capacity (∼9 kb), tissue-targeting capability	Insertional mutagenesis risk, immune response concerns, especially *in vivo*
Integration deficient Lentivirus versus Lentiviruses (IDLVs)	Transient CRISPR-Cas9 delivery	Reduced risk of insertional mutagenesis, while maintaining efficient transduction	Reduced expression longevity compared to integrative LVs; may not suffice for long-term therapies
Gamma Retrovirus (γ-RV)	Stable gene delivery for dividing cells	Persistent gene expression in dividing cells	Limited to dividing cells, insertional mutagenesis risk, low efficiency *in vivo*
Electroporation	Permeabilise cell membranes with electric fields to insert components	Effective for various cell types, applicable to all CRISPR formats (DNA, mRNA, protein)	It can cause cell death, and complex optimisation is required for each cell type
Microinjection	Direct CRISPR component injection into cells under microscopic guidance	Precise control over delivery, minimal off-target effects	Labor-intensive, limited to single-cell applications, requires specialised equipment
Hydrodynamic Injection	Rapid injection for liver-targeted delivery	Effective for a liver-targeting, simple technique	High-pressure risks (e.g., vessel damage, liver stress), limited to liver tissue
Lipid Nanoparticles	Encapsulate nucleic acids, facilitate cellular entry via endocytosis	Biocompatible, allows cell-specific targeting (e.g., hepatocytes)	Risk of endosomal trapping, potential degradation in the bloodstream
Polymer-Based Nanoparticles	Deliver nucleic acids with controlled release	Modifiable for biocompatibility, sustained release	Cytotoxicity at higher doses, complex synthesis, requires extensive optimisation
- Inorganic Nanoparticles	Deliver CRISPR components useful for tracking and imaging	High stability, controlled release, optical properties for tracking	Risk of tissue accumulation, potential long-term toxicity, often not biodegradable
Viral-like Particles	Deliver CRISPR components with lower immunogenicity	High transduction efficiency, lower immune response, customisable targeting	Synthetic peptide limitations, complex production process
Exosomes	Deliver CRISPR with natural cell-derived vesicles	High biocompatibility and low immune response can cross barriers (e.g., blood-brain barrier)	Low production yield, complex isolation and purification, limited targeting without modifications

## 4 Key application of CRISPR-Cas technology in medicine

### 4.1 Genome-editing CRISPR-based therapies: correction of genetic disorders

Genetic disorders are caused by alterations in an individual’s genomic sequence, often resulting from mutations in specific genes. These disorders encompass a broad spectrum of diseases, often inherited from parents to offspring through defective genes. Mutations play a central role in these disorders by producing non-functional or harmful proteins, ultimately causing cellular dysfunction and diseases of various severity (Jackson et al., 2018; Roth and Marson, 2021). The impact of such mutations on human health can be profound, manifesting as chronic illnesses and physical disabilities and often resulting in substantial medical and economic burdens on patients and healthcare systems (Miller et al., 2020). The burden of inherited diseases is substantial, both in terms of human suffering and economic costs. Conditions such as sickle cell disease and thalassemia, for example, impose heavy demands on healthcare resources due to their chronic nature and the need for ongoing treatment (Miller et al., 2020).

CRISPR-Cas technology has emerged as a transformative tool in genetic medicine, paving the way for developing novel therapies for previously untreatable and deadly inherited diseases by providing targeted DSBs for precise gene knockouts, single-stranded breaks, specific nucleotide changes, insertions, or corrections, restoring normal gene function. CRISPR-based somatic gene editing alters non-reproductive cells, ensuring modifications are not inherited, and shows great promise in treating genetic disorders and cancers (Yang et al., 2021; Chanchal et al., 2024). Moreover, research conducted in cell cultures, animal models, and hematopoietic progenitor cells (HPCs) has demonstrated the efficacy of CRISPR-based therapies in correcting mutations and actively restoring normal gene function (Liu et al., 2021). In a study, CRISPR-Cas9 was used to treat β-thalassemia by editing the α-globin locus in human hematopoietic stem/progenitor cells (HSPCs). The strategy combines α-globin downregulation (via *HBA2* gene deletion) and β-globin expression enhancement (through β-globin transgene integration). The approach successfully corrected the pathological phenotype in cellular models and maintained long-term repopulation in xenotransplanted mice. In β-thalassemia patient HSPCs, it corrected the α/β globin imbalance, and Cas9 nickase editing provided a safer, precise alternative (Pavani et al., 2021).

Hemochromatosis, a prevalent inherited metabolic disorder in white populations, often results from a C282Y mutation in the HFE gene, where a G > A mutation at c.845 disrupts HFE protein folding, preventing it from reaching the cell membrane. This absence inhibits interaction with transferrin receptors 1 and 2, leading to iron overload (Fleming et al., 2005). In a study, optimized gRNAs were screened in cell culture, and an AAV8 split-vector delivering adenine base editor ABE7.10 with a specific gRNA was tested in 129-Hfetm.1.1Nca mice. A single injection corrected the mutation in over 10% of cells and improved hepatic iron metabolism, offering a potential gene correction therapy for this common hereditary disease (Rovai et al., 2022).

Cystic fibrosis (CF), a recessive disorder arising from diverse mutations in the cystic fibrosis transmembrane conductance regulator (*CFTR*) gene, affects individuals with mutations unresponsive to *CFTR* modulators, leaving approximately 8% of patients without effective treatments (Cutting et al., 2019). A mutation-agnostic strategy was proposed using CRISPR-Cas9 to insert *CFTR* cDNA with a transcription termination sequence into exon 1 of the *CFTR* locus, enabling the expression of functional *CFTR* mRNA under natural transcriptional regulation. This approach demonstrated safety, with minimal concerns about genomic rearrangements or regenerative decline in edited basal epithelial cells. While the findings support this strategy as a durable therapy for CF, questions remain about the impact of low-level chromosomal aberrations (∼1%) and the challenges of effective cell implantation or targeted *in vivo* delivery to airway epithelial basal cells (Porter and Lueck, 2024).

Alzheimer’s disease is a progressive neurodegenerative disorder characterized by cognitive decline, memory loss, and personality changes, largely due to the accumulation of amyloid-β plaques and tau tangles in the brain (Sheppard and Coleman, 2020). *In vivo* gene editing in adult brain neurons could offer a promising approach for neurological disease treatment. A study developed CRISPR-Cas9 nano-complexes, demonstrating efficacy in the adult mouse brain with minimal off-target activity. Targeting the Bace1 gene, this system reduced amyloid beta (Aβ)-related pathologies and cognitive impairments in two Alzheimer’s disease mouse models, highlighting the broader potential of CRISPR-Cas9 for treating neurodegenerative diseases (Park et al., 2019).

Severe combined immunodeficiency (SCID) encompasses genetic disorders that hinder lymphocyte maturation and function, causing profound immune deficiency (Justiz-Vaillant et al., 2023). A study explored the CRISPR-Cas9 approach to correct SCID-causing mutations by editing the patient’s hematopoietic stem and progenitor cells (HSPCs) *ex vivo*. By engineering SCID-like mutations in healthy donor CD34^+^ HSPCs, researchers developed a model to study gene correction strategies. Using this model, they performed gene correction in *RAG2*-SCID patient-derived HSPCs, producing CD3^+^ T cells with diverse T cell receptors, suggesting the feasibility of restoring immune function through targeted gene editing (Iancu et al., 2022).

Recently, a vast number of genetic disorders have undergone CRISPR-Cas gene therapy, primarily in proof-of-principle studies. Table 2 summarises several diseases, their associated gene mutations, the targets of CRISPR-Cas interventions, and findings of studies. This table offers a detailed overview of current research, focusing exclusively on cases where CRISPR-Cas technology has been employed to directly target and correct genetic mutations. These advancements hold tremendous promise for effectively treating inherited diseases.

**TABLE 2:** CRISPR-Cas-based gene corrections for genetic disorders.

Target organ	Disease	Study target	Findings	Ref no.
Blood	Hemophilia B	*F9*	Corrected *F9* gene in iPSCs using CRISPR-Cas9; restored *F9* expression in hepatocyte-like cells	Morishige et al. (2020)
Blood	Hemophilia A	*F8* (large chromosomal inversions)	Reverted chromosomal inversions in F8 using CRISPR-Cas9; restored F8 function in endothelial cells	Park et al. (2015)
Bone Marrow	Fanconi Anemia	*Fancf*	Corrected *FANCF* mutation with CRISPR-Cas9; improved cell survival and proliferative advantage	Vrugt et al. (2019)
Lung	Cystic Fibrosis	*CFTR*	Corrected *CFTR* mutations in lung cells using SORT LNPs with CRISPR-Cas9; restored CFTR function	Wei et al. (2023)
Lung	α1-Antitrypsin Deficiency	*SERPINA1* (PiZ mutation)	Gene editing via adenovirus in the AATD mouse model corrected the PiZ mutation, improving liver histology and reducing fibrosis	Bjursell et al. (2018)
Liver	Hemochromatosis	*HFE* (C282Y mutation)	AAV8 vector expressing adenine base editor corrected *HFE* mutation in mice, improving iron metabolism	Rovai et al. (2022)
Liver	Familial hypercholesterolemia	*PCSK9*	CRISPR-Cas9 targeting *PCSK9* in hepatocytes reduced LDL-cholesterol levels by 60% in mice and primates	Hoekstra and Van Eck (2024)
Liver	Phenylketonuria (PKU)	*PAH*	AAV2/8 vectors and vanillin co-administered to promote gene correction in PKU mice, partially restoring *PAH* activity and reducing blood phenylalanine levels	Richards et al. (2019)
Liver	Hereditary Tyrosinemia Type I (HTI)	*FAH*	Cas9 nickase gene editing in HTI rats corrected the *Fah* mutation, leading to liver regeneration, weight gain, and survival, with minimal off-target effects	Shao et al. (2018)
Liver	Hereditary Tyrosinemia Type I (HTI)	*FAH*	Base editing corrected a start codon mutation in HTI mice, restoring *Fah* gene expression and improving liver function without causing off-target effects	Yang et al. (2020)
Liver	Glycogen Storage Disease type Ia (GSD-Ia)	*G6PC* (G6PC-p.R83C mutation)	CRISPR-Cas9 editing corrected the G6PC-p.R83C variant in a GSD-Ia mouse model, stabilizing glucose levels and preventing hypoglycemia	Arnaoutova et al. (2021)
Muscle	Duchenne Muscular Dystrophy (DMD)	*DMD*	CRISPR-Cas9-mediated gene editing of the *DMD* gene in all studies targeting different exons proved highly efficient	Long et al. (2014), Nelson et al. (2016), Tabebordbar et al. (2016)
Muscle	Limb-girdle Muscular Dystrophy	*CAPN3* (c.550delA mutation)	CRISPR-Cas9 successfully corrected the *CAPN3* c.550delA mutation in patient-derived iPSCs and primary human muscle stem cells, restoring the wild-type sequence and CAPN3 protein expression	Müthel et al. (2023)
Eye	Leber Congenital Amaurosis Type 10 (LCA10)	*CEP290* (IVS26 mutation)	CRISPR-Cas9 successfully removed a pathogenic splice donor mutation in the *CEP290* gene, restoring normal gene expression in mouse and non-human primate models	(Maeder et al., 2019, p. 10)
Eye	Retinitis Pigmentosa	*Nrl*	CRISPR-Cas9 targeting *Nrl* improved rod survival and preserved cone function in mice with retinal degeneration	Yu et al. (2017)
Eye	Cataracts	*Crygc*	CRISPR-Cas9 corrected *Crygc* mutations in mice via HDR, with rare off-target effects; the corrected mice were fertile and passed on the mutation	Wu et al. (2013)
Kidney	Autosomal Dominant Polycystic Kidney Disease (ADPKD)	*PKD2* (R803X mutation)	CRISPR-Cas9 corrected the R803X mutation in an iPSC line, maintaining normal cell function and differentiation	Liu et al. (2020)
Ear	Deafness	*Atp2b2, Tmc1*	CRISPR-Cas9 editing in mice with *Atp2b2* mutations improved outer hair cell function and hearing; dual-targeting also showed partial hearing recovery	Tao et al. (2023)
Skin	Recessive dystrophic epidermolysis bullosa (RDEB)	*COL7A1*	New nonviral carriers delivered CRISPR-Cas9 to delete *COL7A1* exon 80 mutation, restoring type VII collagen production	Wang et al. (2023b)
Nervous System	Amyotrophic lateral sclerosis (ALS)	*SOD1*	CRISPR-Cas9 reduced mutant *SOD1* levels in mice, improving motor function and extending survival	Gaj et al. (2017)
Nervous System	Alzheimer’s disease (AD)	*ApoE4*	CRISPR-Cas9 specifically knocked out ApoE4 in mice, reducing protein levels by 70% with no effect on *ApoE3*	Rabinowitz et al. (2019)
Nervous System	Parkinson’s disease (PD)	*Tyrosine hydroxylase (th)*	SAM system activated endogenous tyrosine hydroxylase in astrocytes, producing dopamine and improving motor function in rats	Narváez-Pérez et al. (2024)
Nervous System	Neurofibromatosis type 1 (NF1)	*Nf1*	CRISPR-Cas9 generated a rat model with allele-specific neurofibromin truncation, revealing pain mechanisms and potential therapeutic targets	Moutal et al. (2017)
Nervous System	Fragile X syndrome (FXS)	*FMR1*	CRISPR-Cas9 was utilised to excise the expanded CGG-repeat; transcriptional reactivation was observed in 67% of hybrid colonies and 20% of iPSC colonies, with FMRP production and reduced DNA methylation	Xie et al. (2016)
Immune system	Chronic granulomatous disease (CGD)	*CYBB*	CRISPR-Cas9 corrected CYBB intronic mutations in iPSCs, and restored oxidative burst function in phagocytes, demonstrating a viable gene therapy approach	Flynn et al. (2015)
Immune System	Severe combined immunodeficiency (SCID)	*RAG2*	CRISPR-Cas9 edited CD34^+^ HSPCs from healthy donors to model SCID and therapeutic outcomes; successful gene correction in *RAG2*-SCID patient-derived HSPCs with diverse TCR repertoires	Iancu et al. (2022) and Brault et al. (2023)
Immune System	Severe combined immunodeficiency (SCID)	*IL2RG*	CRISPR-Cas9-based targeted insertion (TI) of *IL2RG* in HSPCs showed superior NK cell development and reduced off-target effects compared to the lentivector (LV) approach	
Immune System	Wiskott-Aldrich syndrome (WAS)	*WAS*	CRISPR-Cas9 corrected WAS mutations in HSPCs with up to 60% efficiency, restored WASp expression, and showed no major genotoxicity in transplanted mice	Rai et al. (2020)
Systemic diseases	Hutchinson-Gilford progeria syndrome (HGPS)	*LMNA*	Adenine base editors (ABEs) corrected pathogenic HGPS mutations in fibroblasts and mice, improved lifespan, and improved vascular pathology, showing promise for *in vivo* base editing	Koblan et al. (2021)

### 4.2 Epigenome-editing CRISPR-based therapies

The epigenome encompasses all the chemical modifications to DNA and histone proteins within a cell, playing a crucial role in regulating gene activity, maintaining cellular identity, and facilitating dynamic responses to environmental stimuli. In normal physiological conditions, the epigenome orchestrates the intricate regulation of gene expression, ensuring that genes are activated or silenced in a context-specific manner (Hamilton, 2011). DNA methylation typically occurs at cytosine residues within CpG dinucleotides and is associated with transcriptional repression (Jin et al., 2011). Histone modifications, such as acetylation, methylation, and phosphorylation, can either promote or inhibit gene expression depending on the specific nature and context of the modification (Bannister and Kouzarides, 2011). Additionally, non-coding RNAs, including microRNAs and long non-coding RNAs, contribute to the regulation of gene expression by interacting with mRNA and chromatin-modifying complexes (Bannister and Kouzarides, 2011; Wei et al., 2017).

Epigenome editing refers to the targeted modifications of epigenetic aspects, including DNA methylation, histone modifications, and chromatin remodelling, without changing the DNA sequence. Applying this technique broadens the therapeutic potential of CRISPR-Cas systems beyond traditional gene editing, providing new therapeutic options for diseases linked to aberrant epigenetic changes.

CRISPR-Cas technology has significantly advanced the field of epigenome editing, offering targeted interventions across various diseases. For neurological disorders, enzymes such as dCas9-DNMT3A and dCas9-Tet1 are employed to modulate gene expression in conditions like Parkinson’s disease and Fragile X syndrome, resulting in improved neuronal function and gene regulation (Kantor et al., 2018; Liu et al., 2018). CRISPR-Cas9 targets the *BCL11A* enhancer to adjust fetal haemoglobin levels in haematological disorders, presenting a novel therapeutic strategy (Canver et al., 2015). Cardiac diseases benefit from dCas9-KRAB’s ability to repress the *CALM2* gene in long-QT syndrome, normalising cardiac cellular functions (Limpitikul et al., 2017). In musculoskeletal diseases, dCas9-KRAB and dCas9-VP64 address inflammatory signalling and muscle dystrophies by targeting relevant genes such as *TNFR1/IL1R1* and *Lama1* (Farhang et al., 2017). Pulmonary and metabolic disorders are targeted with dCas9-Dnmt3A and dCas9-ATS-9R, respectively, showing potential for reversing gene overexpression and managing metabolic conditions (Qu et al., 2018; Chung et al., 2019). Liver diseases, including cancer, are treated using dSaCas9-KRAB and dCas9-cre-CRISPRa systems, which target genes like *Pcsk9* and proto-oncogenes, offering advances in cholesterol management and cancer genetics (Thakore et al., 2018; Wang et al., 2018a; Wangensteen et al., 2018). Retinal diseases benefit from dCas9-KRAB’s role in converting rod cells into cone-like cells, while autoimmune conditions are managed by dCas9-VP160 and CRISPR-Cas9 systems targeting inflammatory pathways and gene reactivation (Jing et al., 2015; Giménez et al., 2016; Moreno et al., 2018). Various cancers, including prostate, liver, colon, lung, breast, melanoma, and bladder, are addressed using different CRISPR-Cas systems to suppress oncogenes, activate tumour suppressors, and modulate regulatory RNAs.

The advancements in CRISPR-Cas technology for epigenome editing have profoundly impacted the field of medicine by offering precise tools for modulating gene expression while avoiding the need for direct DNA sequence alterations. This approach minimises the risks associated with permanent genetic modifications and provides a promising avenue for therapeutic interventions across various diseases. For detailed descriptions of these applications and their outcomes, refer to Table 3. This table provides an in-depth look at how CRISPR-Cas systems target specific epigenetic modifications and their therapeutic potential across various diseases.

**TABLE 3:** Epigenetic editing by different Cas variants in different diseases.

Target organ/Disease	Epigenome editor	Study target	Findings	Ref.
Neurological disorders	dCas9- DNMT3A	*SNCA* gene in Parkinson’s disease	Precise reduction in *SNCA* mRNA and protein levels, leading to the restoration of neuronal phenotypes associated with the disease	Kantor et al. (2018)
Neurological disorders	dCas9-Tet1	*FMR1* gene in Fragile X syndrome	Reversed hypermethylation and subsequent restoration of *FMR1* expression, improving neuronal phenotypes	Liu et al. (2018)
Neurological disorders	CRISPR-Cas9	*UBE3A* gene in Angelman syndrome	Reactivation of the paternal *UBE3A* gene, leading to improvements in behavioural phenotypes	Wolter et al. (2020)
Hematological disorder	CRISPR-Cas9	*BCL11A* enhancer for fetal haemoglobin	*BCL11A* enhancer validated as a therapeutic target for genome editing in β-hemoglobin disorders	Canver et al. (2015)
Cardiac disease	dCas9-KRAB	*CALM2* gene in long-QT syndrome	Selective repression of the mutant *CALM2* gene, resulting in the normalisation of cellular function	Limpitikul et al. (2017)
Musculoskeletal Diseases	dCas9-KRAB	*TNFR1/IL1R1* in inflammatory signalling	Enhanced cell survival and differentiation under inflammatory conditions	Farhang et al. (2017)
Muscle atrophy	CRISPR-Cas9	miR-29b	Mitigated muscle atrophy and improved exercise capacity in mice	Li et al. (2020)
Neuromuscular disorders	dCas9-VP64	*Lama1* gene in MDC1A	Upregulated *Lama1* expression prevented muscle fibrosis and paralysis, even reversing symptoms in affected mice	Kemaladewi et al. (2019)
Muscular dystrophy	dCas9-VP160	*Lama1* gene	Significant upregulation of Lama1 in muscle tissues, offering a potential therapeutic approach for myopathies	Perrin et al. (2017)
Pulmonary Disease	dCas9-Dnmt3A	*DSP* gene	Reversed overexpression of *DSP* via targeted DNA methylation	Qu et al. (2018)
Metabolic Disease	dCas9-ATS-9R	*Fabp4* gene	Reduced body weight, inflammation, and hepatic steatosis in a mouse model	Chung et al. (2019)
Metabolic Disease	dCas9-VP64	*Sim1* and *Mc4r* genes	Restored obesity phenotype in haploinsufficient mice	Matharu et al. (2019)
Liver Disease	dSaCas9-KRAB	*Pcsk9* gene	Achieved long-term reduction in serum Pcsk9 and cholesterol levels	Thakore et al. (2018)
Retinal Disease	dCas9-KRAB	*Nrl* gene	Converted rod cells into cone-like cells, preventing cone cell loss	Moreno et al. (2018)
Autoimmune Disease	dCas9-VP160	*INS* gene	Successfully reactivated the silenced human *INS* gene in fibroblasts derived from T1D patients without altering DNA methylation	Giménez et al. (2016)
Autoimmune Disease	CRISPR-Cas9	miR-155	Generated a miR-155 knockout macrophage cell line, which decreased proinflammatory cytokine production	Jing et al. (2015)
Multisystem Disorders	Cas9-MS2-P65-HSF1 (MPH)	Various genes	Enabled *in vivo* gene activation, leading to symptom improvement in mouse models	Liao et al. (2017)
Prostate Cancer	dCas9-KRAB	*PSA* gene	Suppression of *PSA* gene expression, leading to reduced tumour growth and migration, and promotion of apoptosis in prostate cancer cells	Yang et al. (2023)
Colon Cancer	dCas9-TET1CD	SARI promoter	Targeted demethylation reactivated SARI expression, exerting antitumour effects by modulating cell proliferation and apoptosis	Wang et al. (2019c)
Lung Cancer	dCas9-DNMT3a	SMARCA2 promoter	Promoter hypermethylation resulted in the downregulation of SMARCA2, highlighting its role as a tumour suppressor in lung cancer	Wu et al. (2019)
Lung Cancer	dCas9 with effector domains (VP64, p300, VPR, SAM)	*MASPIN* and *REPRIMO* tumour suppressor genes	Combined CRISPR-dCas9 with multiple effector domains effectively reactivated *MASPIN* and *REPRIMO* genes, inhibiting cell proliferation and inducing apoptosis	Garcia-Bloj et al. (2016)
Breast Cancer	dCas9-DNMT3a	*CDKN2A, RASSF1, HIC1, PTEN*	*De novo* DNA methylation led to the repression of CDKN2A, preventing cellular senescence and promoting tumour initiation	Saunderson et al. (2017)
Breast Cancer	CRISPR-Cas9	miR-3662 microRNA	Inhibition or knockout of miR-3662 reduced TNBC cell proliferation and migration, decreasing tumour growth and metastasis. This had an impact on Wnt/β-catenin signalling	Yi et al. (2022)
Melanoma and TNBC	dCas9-VPR	*PTEN* gene	Activation of *PTEN* by dCas9-VPR restored its expression, suppressed oncogenic pathways, and reduced tumour growth and migration, particularly in BRAF mutant melanoma	Moses et al. (2019)
Bladder cancer	dCas9-VP64	KLF4, a transcription factor associated with carcinogenesis	Overexpression of KLF4 inhibited tumourigenesis and epithelial-mesenchymal transition (EMT) in UBC cells, suggesting its potential therapeutic application	Xu et al. (2017)
Medulloblastoma	dCas9-VP160	*MYC* gene	Induced MYC expression resulted in large cell anaplastic medulloblastomas, providing a model for pre-clinical studies of MYC transcription regulation	Vo et al. (2018)
Glioblastoma	CRISPR-Cas9	miR-10b microRNA	Knockout of miR-10b proved lethal to glioma cells, eliminating tumour growth and neoplastic transformation, highlighting miR-10b as a critical regulator of GBM.	Fatimy et al. (2017)

### 4.3 Applications of CRISPR-Cas technology in oncology

#### 4.3.1 Advancing CAR-T and NK cell immunotherapies

One prominent application of CRISPR-Cas9 technology is its application in engineering T-cells express CARs. CAR-T cell therapy is a genetically modified T-cell that expresses CARs, targeting tumour-associated antigens (TAAs) or tumour-specific antigens (TSAs) with high specificity, thereby targeting and eliminating cancer cells (Jogalekar et al., 2022). The therapy involves harvesting T cells from the patient’s blood, genetically engineering them to express CARs, expanding them *ex vivo*, and reinfusing them into the patient. CRISPR-Cas9 technology has enhanced CAR-T therapy by enabling precise genetic edits that improve T cell functionality, persistence, and specificity (Dimitri et al., 2022). For example, CRISPR-mediated knockout of immune checkpoint molecules, such as programmed cell death protein 1(PD-1), demonstrated promising antitumor activity of CAR-T cells by inhibiting their exhaustion and enhancing persistence in the hostile tumour microenvironment (TME) (Yan et al., 2024). Similarly, deleting (*CTLA-4*) in T-cells significantly enhanced their anti-tumour activity through increased secretion of TNF-α and IFN-γ (Zhang et al., 2019).

Targeted knock-in at loci such as T Cell Receptor Alpha Constant (*TRAC*) allows stable CAR insertion without the risk of random integration and the secondary effects such as clonal expansion, oncogenic transformation, and diversified CAR expression associated with viral-mediated transfection. Studies have shown that CAR-T cells engineered through CRISPR-mediated knockin at T-cell receptor alpha constant (*TRAC*) loci exhibit consistent CAR expression, enhanced anti-tumour efficacy and persistence (Eyquem et al., 2017).

CRISPR-Cas9 has enabled the generation of universal CAR-T cells by allowing precise genetic modifications to reduce alloreactivity and make CAR-T cells more suitable for off-the-shelf use. The process involves editing key genes, such as *TRAC* and T-cell receptor beta constant 1 (*TRBC*), which encode the TCR chains, to create TCR-knockout CAR-T cells. This disruption prevents graft-versus-host disease (GVHD). Additionally, strategies such as beta-2-microglobulin *(B2M)* knockout can further reduce alloreactivity by eliminating major histocompatibility complex (MHC) class I expression. Multiplexed knockout of *TRAC*, *B2M*, and PD-1 in CD19-specific CAR-T cells demonstrated enhanced anti-tumor activity and reduced alloreactivity, offering promise for more accessible, universal CAR-T therapies (Stenger et al., 2020; Song et al., 2024).

Cancer immunotherapy requires effective immune cell trafficking and infiltration into the tumour tissue. An effective strategy to enhance this process is to engineer and modify immune cells with specific chemokines or their receptor receptors, which significantly direct and control cell migration to specific tissue sites (Du et al., 2022). For instance, engineered T-cells expressing higher levels of c-c motif chemokine ligand 19 (*CCL19*), a chemokine ligand, significantly improved CAR-T cell tumour infiltration and survival (Adachi et al., 2018).

Beyond CAR-T cells, CRISPR-Cas9 technology is being used to modify and improve the efficacy and persistence of CAR-expressing natural killer (CAR-NK) cells for cancer immunotherapy. In a study, CRISPR-Cas9 was utilised to upregulate CXC chemokine receptor 2 (*CXCR2*) and interleukin-2 (*IL-2*) expression on NK-92 cells. The upregulation of *CXCR2* and *IL-2* was demonstrated to enhance NK-92 cell migration to tumour sites, improve cell proliferation, and increase cytotoxicity. Additionally, these gene-edited NK-92 cells exhibited greater inhibition of human colon cancer growth *in vivo*, reducing tumour burden and a marked extension of survival time in tumour-bearing mice (Gao et al., 2021).

Further studies showed that disrupting the natural killer group 2 member a (NKG2A) gene, killer cell lectin like receptor C1 (*KLRC1*), which encodes an inhibitory receptor on NK cells that often limits their tumour-killing ability through interaction with HLA-E on acute myeloid leukaemia (AML) cells, on CD33-specific CAR-NK cells demonstrated enhanced resistance to immune suppression. Moreover, these gene-edited CAR-NK cells showed increased anti-cancer activity, demonstrating significant cytotoxic effects against AML in laboratory and animal models (Bexte et al., 2024).

To minimise immune rejection and enhance compatibility in NK cell therapies, CRISPR-Cas9 was used to knock out the B2M gene, eliminating the surface expression of HLA class I molecules. A single-chain HLA-E molecule was co-expressed to prevent NK cell fratricide from “missing self” signals. These engineered NK cells retained functional characteristics, including cytotoxicity against AML cell lines, without activating allogeneic T cells. This modification supports the feasibility of using non-HLA-matched, “off-the-shelf” NK cells for cancer immunotherapy (Hoerster et al., 2021).

CRISPR-Cas9 technology has been used to reprogram immune cells’ intrinsic capabilities. For example, CRISPR-Cas9 was utilised to engineer macrophages with signal regulatory protein alpha (*SIRP-α*) knockout, eliminating the “do not eat me signal”. This modification has four-fold enhanced the phagocytic capacity of the macrophages (Ray et al., 2018). Integrating CRISPR-Cas technology into cancer immunotherapy development techniques holds significant potential for successfully treating cancerous diseases and achieving more efficient and personalised therapeutic approaches.

#### 4.3.2 Modifying oncogenes and tumour suppressor genes

Oncogenes, which are the mutated or overexpressed proto-oncogenes, contribute to tumourigenesis by driving uncontrolled cell proliferation and survival (Kontomanolis et al., 2020). In contrast, tumour suppressor genes (TSGs) usually prevent cancer by regulating cell growth and promoting apoptosis. They require biallelic inactivation for tumourigenesis; their mutation or deletion results in uncontrolled cell division and cancer development (Wang et al., 2018b).

CRISPR-Cas9 disrupts oncogenes or repairs mutations in tumour suppressor genes, restoring their function and providing targeted interventions in cancer treatment. The *MYC* gene is frequently amplified and overexpressed in various cancers, including breast, lung, and colorectal. Knocking out MYC oncogenes has been shown to reduce cell proliferation in animal models (Chehelgerdi et al., 2024). Another application is the potential reactivation of tumour suppressor genes, like tumor protein p53 (*TP53*), which is often mutated in many cancers. CRISPR-Cas9 could be utilised to correct *TP53* mutations in cancer, restoring its normal function, which induces cell cycle arrest and apoptosis in cancer cells (Mirgayazova et al., 2020). In another instance, corrections of kirsten rat sarcoma viral oncogene homolog (*KRAS*) G12D mutation, observed in many cancers, showed reduced cell growth compared to the wild-type cells with *KRAS* G12D mutations (Lentsch et al., 2019).

CRISPR-Cas technology has also been effective in targeting viral oncogenes, which are highly implicated in the development and progression of several cancer types. An impressive application is a direct disruption of viral DNA integrated into the host genome, thereby inhibiting oncogene expression and inhibiting tumourigenesis (Oppel et al., 2018). This method has shown promising results in the context of human papillomavirus (HPV)-related cancers. HPV is a significant cause of the development of head and neck squamous cell carcinomas (HNSCCs) and cervical cancer, primarily by expressing human papillomavirus oncoprotein e6 and e7 (*E6* and *E7*) oncogenes (Szymonowicz and Chen, 2020). The E6 protein induces the tumour suppressor protein p53 degradation, inhibiting cell apoptosis and enhancing uncontrolled cellular proliferation. Simultaneously, the E7 protein inactivates the retinoblastoma protein (*pRb*), impairing cell cycle control and further contributing to oncogenesis (Janiszewska et al., 2024). In a study, targeted knockdown of the promoter region of HPV-16 *E6* and *E7* oncogenes, or the transcripts by CRISPR-Cas9, achieved significant therapeutic outcomes (Zhen et al., 2014). This method significantly reduced the proliferation of cervical cancer cells *in vitro*, which was correlated with elevated levels of p53 and p21 protein. Moreover, transplanting these edited cells into nude mice resulted in substantially declining tumour formation and growth (Zhen et al., 2014). Another study revealed that the *in-vivo* delivery of the CRISPR-Cas9 complex via PEGylated liposomes eradicated cancer cells and showed complete survival of the HPV-induced cervical cancer animal model (Jubair et al., 2019).

These findings demonstrate the effectiveness of CRISPR-Cas9 technology as a therapeutic approach for treating HPV-related malignancies and a promising method for eliminating viral oncogenes.

#### 4.3.3 CRISPR-based oncolytic viruses

Virotherapy is a novel approach to cancer treatment based on the ability of oncolytic viruses (OVs) to lyse cancer cells. OVs are genetically modified viruses that selectively lyse and destroy malignant cells while sparing normal tissues (Lin et al., 2023). Manipulating viral genomes, especially for oncolytic herpes simplex viruses (oHSVs), has classically relied on laborious methods involving homologous recombination via bacterial artificial chromosomes (BACs). Luckily, the emergence and development of CRISPR-Cas9 technology has significantly enhanced this process. CRISPR-Cas gene editing is utilised to introduce oncolytic viruses with therapeutic genes, enhancing their cancer tissue selectivity and suppressing antiviral protective mechanisms employed by malignant cells (Wang et al., 2022b). In one study, the Herpes Simplex Virus type 1 (HSV-1) genome was altered using CRISPR-Cas9 to replace the herpes simplex virus 1 protein icp34.5 (*ICP34.5*) coding area with murine interleukin 12 (*IL12*) and CXC motif chemokine ligand 11 (*CXCL11*), and to delete the herpes simplex virus 1 protein icp47 (*ICP47*) gene. These genetic alterations markedly reduced viral pathogenicity and increased tumour selectiveness. The results showed great promise for treating colorectal cancer (CRC) (Zhang et al., 2023b).

Other variants of HSVs have been engineered using CRISPR-Cas9. *ICP6*-mutated Herpes Simplex Viruses (HSVs), including rHSV1/∆RR (with a deletion of the ribonucleotide reductase ICP-6) and rHSV1/∆ICP6 (a complete deletion) revealed potent cytotoxicity and selectivity for targeting tumour cells from *in-vitro* studies. Similarly, *in-vivo*, they demonstrated reduced pathogenicity while substantially reducing tumour burden and improved survival rates. The rHSV1/∆RR variant, notably, elicited a robust antitumor immune response, marked by increased neutrophil infiltration and elevated levels of antitumor cytokines (Ni et al., 2022).

Leveraging the CRISPR-Cas9-assisted recombinant vaccinia virus engineering (CARVE) system, a recombinant vaccinia virus, STINGPOX, has been successfully engineered, expressing three distinct transgenes at separate genomic loci. A transgene encodes a bacterial diadenylate cyclase, which synthesises cyclic di-AMP—a potent agonist of the stimulator of interferon genes (*STING*) pathway. This agonist effectively stimulates interferon (IFN) signalling, a critical component of the antitumor immune response. The ability of STINGPOX to induce IFN signalling was confirmed in primary human cancer tissue explants. Furthermore, when STINGPOX was combined with the checkpoint inhibitor anti-PD-1, it significantly increased post-cancer survival in an immunocompetent mouse model of colon cancer (Whelan et al., 2023). These developments demonstrate the exceptional capacity and promising efficacy of CRISPR-based oncolytic viruses to target cancer cells precisely.

#### 4.3.4 Tumour microenvironment and drug resistance

The TME is a network surrounding the tumour that includes stromal cells, extracellular matrix (ECM) proteins, immune cells, blood vessels, and soluble factors. These components work together to dynamically regulate tumorigenesis and therapeutic responses (Anderson and Simon, 2020).

Stromal cells, such as myofibroblasts and cancer-associated fibroblasts (CAFs), play important roles by altering the ECM and secreting substances that promote the growth and invasion of tumour cells (Guo and Xu, 2024). Malignancy frequently triggers pathological changes to the ECM, which can change tissue stiffness and affect the movement of tumour cells, making drug delivery complicated (Winkler et al., 2020). Tumour-associated macrophages (TAMs), another cell type found in the TME, can either promote the growth of tumours via immune suppression and angiogenesis or obstruct anti-tumour immunological responses, thereby affecting the efficacy of immunotherapies (Pan et al., 2020). The formation of new blood vessels, driven by factors like VEGF, results in a disorganised vascular network that impedes effective drug penetration (Lugano et al., 2019). Additionally, soluble factors such as cytokines and growth factors modulate the TME’s impact on drug efficacy and immune interactions (Zhang et al., 2010). These factors contribute to therapeutic resistance by affecting drug distribution, metabolism, and immune surveillance, presenting significant challenges for cancer treatment strategies (Wang et al., 2023a).

CRISPR-Cas9 technology and novel delivery systems have emerged as a powerful tool for elucidating and manipulating the TME to improve therapeutic outcomes. In a study to address challenges in delivering gene-editing tools to solid tumours, a multiplexed dendrimer LNP system was developed to co-deliver focal adhesion kinase (*FAK*) siRNA, Cas9 mRNA, and sgRNA to tumours, enhancing gene editing efficiency. This system, siFAK + CRISPR-LNPs, improved gene editing in tumour spheroids over ten-fold through higher cellular uptake and penetration by FAK knockdown. This method also diminished ECM stiffening and effectively inhibited programmed death-ligand 1 (*PD-L1*) expression via CRISPR-Cas9 to dramatically suppress tumour growth and metastasis in 4 mouse cancer models (Zhang et al., 2022).

The CRISPR-Cas9 system is applied to modify CAFs inside the TME through targeting fibroblast activation protein (*FAP*) and C-X-C Motif Chemokine Ligand 12 (*CXCL12*). Abrogating these genes potentially reduces the pro-tumourigenic impact of CAFs by reducing collagen deposition and mitigating their ability to recruit immunosuppressive cells (Kalluri, 2016). Ultimately, this strategy may render the TME less pro-tumorigenic and more accessible to effective therapeutic intervention (Zhang et al., 2024).

CRISPR-Cas9 technology is increasingly utilised to address the mechanisms of cancer drug resistance by targeting resistance pathways, specifically, the genes involved in drug metabolism, efflux pumps, or resistance-associated mutations. A study utilised CRISPR-Cas9 to disrupt the function of *ABCB1* (ATP-Binding Cassette Subfamily B Member 1). This gene encodes P-glycoprotein, a prominent efflux pump responsible for drug extrusion from cancer cells. Inhibiting the expression of *ABCB1* can reduce drug efflux and thereby increase the intracellular concentration of chemotherapeutic agents, enhancing their efficacy (Yang et al., 2016). Studies have demonstrated that CRISPR-Cas9-mediated knockout of *ABCB1* in various cancer cell lines increases sensitivity to several drugs (Zhang et al., 2023a).

*TP53* mutations are frequently linked to cancer drug resistance. These *TP53* mutations are corrected using CRISPR-Cas technology, inhibiting associated pathways leading to drug resistance. For example, research has shown that restoring normal apoptotic responses through CRISPR-Cas9-mediated restoration of wild-type *TP53* in cancer cells can desensitise them to chemotherapy and radiation therapy (Wiegering et al., 2017; Tang et al., 2019). Despite still being in preclinical stages, using CRISPR-Cas9 to manipulate the TME and address cancer drug resistance has shown great promise as a novel approach to cancer treatment.

### 4.4 CRISPR in infectious disease management

#### 4.4.1 CRISPR-Cas-based antiviral therapies

Since its discovery as a gene editing tool, CRISPR-Cas9 has long been thought to be designed as an effective antiviral therapy. The CRISPR-Cas9 system has been investigated extensively for human immunodeficiency virus 1 (HIV-1) cure. One of the effective strategies involves designing gRNAs targeting the long terminal repeat (LTR) regions of the HIV-1 genome. LTRs are important in viral replication and persistence (van Opijnen et al., 2004).

*In vitro* human T-cell studies demonstrated that CRISPR-Cas9 could excise proviral DNA, leading to a marked reduction in viral replication (Ebina et al., 2013). In a study, the HIV RNA genome targeted with Cas13 significantly reduced viral RNA and impeded viral replication (Yin et al., 2020). Cas13 eliminated the current and latent HIV infection in CD + T-cells (Nguyen et al., 2021).

Additional methods have been employed. One such approach is targeting the CCR5 co-receptor. CCR5 co-receptor represents the key entry points for HIV-1 into CD4^+^ T cells (Faivre et al., 2024). In a study, CRISPR-Cas9 mediated knockout of CCR5 combined with an HIV fusion inhibitor, C46, proved effective for preventing viral infection in the MT4CCR5 cell line (Khamaikawin et al., 2024). This technique gained significant attention since the “Berlin patient” case and his functional cure of HIV following a bone marrow transplant from a CCR5 Δ32 donor, a CCR5 impairing deletion (Gupta et al., 2019).

The CRISPR-Cas9 system can be engineered to introduce site-specific DSBs within the Hepatitis B Virus (HBV) genome, leading to the disruption of essential viral genes such as the S, C, and X genes, thereby inhibiting viral replication (Karimova et al., 2015; Rawal et al., 2024). Moreover, CRISPR-Cas systems have been designed to induce the excision of covalently closed circular DNA (cccDNA), the persistent viral reservoir in infected hepatocytes, which represents a major challenge in achieving a complete cure (Ramanan et al., 2015; Li et al., 2017). Recent studies have shown that CRISPR-mediated editing of HBV DNA reduces viral load and diminishes the expression of viral antigens, including hepatitis B surface antigen (HBsAg), which is crucial for immune evasion (Ramanan et al., 2015). While clinical trials are not yet underway, the preclinical success suggests that CRISPR-Cas9 could eventually become a therapeutic option for chronic HBV infection.

CRISPR-Cas9 has also been investigated as a potential therapeutic tool for human papillomavirus (HPV), particularly high-risk strains like HPV16 and HPV18, which are closely associated with cervical cancer. The approach involves targeting the viral oncogenes E6 and E7, responsible for transforming infected cells into cancerous ones (Tornesello et al., 2020). *In vitro* studies using cervical cancer cell lines have demonstrated that CRISPR-Cas9 can disrupt these oncogenes, leading to apoptosis of the infected cells and a reduction in tumour growth (Zhen et al., 2023). These findings have been corroborated *in vivo* using mouse models, where CRISPR-Cas9 treatment has shown efficacy in reducing the size of HPV-associated tumours (Jubair et al., 2019; Gao et al., 2022).

Finally, the emergence of SARS-CoV-2, the virus responsible for COVID-19, has spurred significant interest in using CRISPR technologies as antiviral strategies. In contrast to other examples, RNA-targeting CRISPR-Cas13 have been studied to cleave the RNA genome of severe acute respiratory syndrome coronavirus 2 (SARS-CoV-2). Degradation of specific SARS-CoV-2 RNA by CRISPR-Cas13 has decreased viral replication in cell culture models. This RNA-targeting strategy will be particularly potent for RNA viruses like SARS-CoV-2, offering a rapid and accurate approach to antiviral intervention (Fareh et al., 2021; Wang et al., 2021). While still primarily in preclinical stages, these studies collectively highlight CRISPR’s vast potential in combating viral infections by directly targeting viral genomes, essential replication genes, or cellular receptors crucial for viral entry, showing promising results against viruses that have long challenged conventional therapeutic approaches.

#### 4.4.2 Combating antimicrobial resistance

Antimicrobial resistance (AMR) poses an important threat to worldwide health, as disseminating resistant bacterial, viral, fungal, and parasitic strains reduces the effectiveness of currently accessible antimicrobial treatments (Salam et al., 2023). Longer hospital stays, increased medical expenses, and rising mortality rates are all consequences of AMR (de Kraker et al., 2016). Each year, the AMR in the European Union causes nearly 33.000 fatalities, with €1.5 billion for medical payments because of lack of therapeutic effect and more extended hospital stays (Aljeldah, 2022).

The recent advancements in CRISPR-Cas technology applications in pathogenic bacteria and other microorganisms focus on selective targeting and removal of antimicrobial resistance genes (ARGs). CRISPR-Cas gRNAs targeting the ARGs in different microbial species have been designed to restore these pathogens’ susceptibility to antibiotics (Tao et al., 2022). In a study, the plasmid-encoded Cas9 and sgRNAs could effectively remove native plasmids that contain the MCR-1 gene and MDR genes in clinical isolates of *Escherichia coli* (*E. coli*) (Wang et al., 2019b). In another study, several IS26-based CRISPR-Cas9 plasmids were engineered to target and eliminate specific antibiotic resistance (MCR-1, blaKPC-2, and blaNDM-5) and plasmid replication genes (IncX4, IncI2, and IncHI2) from different plasmids in various *E. coli* strains. CRISPR-Cas9 effectively eliminated these plasmids, inhibited the acquisition of exogenous resistant plasmids, and rendered the bacteria (*E. coli* isolates) susceptible to antibiotics (He et al., 2021). Moreover, the *E. coli* extended-spectrum beta-lactamase (ESBL) gene was targeted by CRISPR-Cas9. By identifying conserved regions in TEM (Temoniera) and SHV (Sulfhydryl variable) type ESBLs across numerous strains, researchers were able to use the CRISPR-Cas9 system to re-sensitise MDR cells by disrupting the plasmid harbouring both target and non-target resistance genes. This approach, termed Re-Sensitization to Antibiotics from Resistance (ReSAFR), underscores the potential of CRISPR-Cas9 to reactivate the efficacy of existing antibiotics (Kim et al., 2016). In a highly innovative study, a broad-host-range IncP1 plasmid pKJK5 was used to introduce a Cas9-containing plasmid targeting AMR genes. This plasmid efficiently prevented the uptake of plasmids containing AMR genes and eliminated an array of *E. coli* and *Pseudomonas* isolates resident plasmids. This study represents an important breakthrough in AMR intervention, emphasising the possibility of CRISPR-Cas approaches targeting a broad spectrum of bacterial species within complicated microbial ecosystems (Walker-Sünderhauf et al., 2023).

Besides plasmid vectors, a different method involves utilising temperate phages to deliver CRISPR-Cas systems directly into the genomes of antibiotic-resistant bacteria. This approach eliminated genetically engineered lytic phages and resistance-conferring plasmids. These phages could be applied as hand and surface cleaners in hospitals to eradicate resistant pathogens while preserving antibiotic-sensitive bacteria electively (Yosef et al., 2015). These investigations indicate the innovative capability of the CRISPR-Cas approach in addressing the AMR crisis by targeting and eliminating the resistance genes, recovering antibiotic susceptibility, and providing innovative, targeted antimicrobial approaches.

### 4.5 CRISPR-Cas application in drug-target discovery and development

CRISPR-Cas technology, particularly CRISPR-Cas9, has profoundly impacted drug discovery, high-throughput screening, and drug-target validation by enabling precise and efficient genetic modifications in mammalian cells. The CRISPR-Cas system’s programmability and adaptability allow it to target virtually any genomic sequence, making it a powerful tool for creating disease models, conducting functional genomics studies, and identifying novel drug targets (Shalem et al., 2015). Through targeted genome editing, CRISPR-Cas facilitates the generation of isogenic cellular and animal models with specific gene mutations, which serve as important platforms for understanding the genetic basis of diseases. Also, it allows researchers to observe the effects of genetic diversity in patients and provide insights into disease mechanisms (Ravichandran and Maddalo, 2023). For instance, cystic fibrosis (CF) is often caused by a specific mutation, ∆F508, in the CFTR gene. Using CRISPR, researchers can create isogenic models of CF patient cells by introducing the ∆F508 mutation into healthy cells or correcting it in patient cells (Schwank et al., 2013). These models allow researchers to screen drugs in the exact genetic environment causing the disease, helping identify compounds that effectively restore CFTR function in ∆F508-mutant cells. Such tailored drug testing is critical because CF patients can have different mutations, which may respond differently to various therapies (Veit et al., 2016).

Genetic perturbations enable a comprehensive understanding of gene-phenotype relationships and the gene-disease pathway mechanism on a large scale, which is pivotal for identifying potential therapeutic targets. By employing libraries of sgRNAs that target thousands of genes across the genome, CRISPR-based screening can reveal genes essential for cell survival, drug resistance, or sensitivity, especially in cancer cells (Bock et al., 2022). In a study, *in-vivo* CRISPR-genome-wide screening was used to identify vulnerabilities in triple-negative breast cancer (TNBC), identifying critical roles for the mTOR and Hippo pathways. The study showed that blocking mTORC1/2 and YAP, an oncoprotein, significantly suppressed tumour growth. Molecular analysis revealed that mTOR inhibition enhances the cancer-killing effect of YAP inhibition, highlighting a promising dual-target strategy for TNBC treatment (Dai et al., 2021).

Genome editing with CRISPR has been used to create model organisms and organoids. In model organisms, this approach allows for studying the disease pathology in CRISPR-edited model organisms, such as mice and zebrafish, that mimic various human diseases, often down to the molecular level, or testing potential therapeutics in those species under physiologically relevant conditions (Hwang et al., 2013). This method dramatically influenced preclinical research, particularly in oncology and rare genetic disorders, where these model organisms are used for testing new potential drug candidates along with efficacy and safety evaluations (Singh and Schimenti, 2015).

Organoids, also known as “mini-organs,” are three-dimensional cellular structures that resemble real organs in architecture and function. They are derived from stem cells, which differentiate into various cell types found in native tissue after developing in a supporting matrix (Schutgens and Clevers, 2020). The advent of CRISPR-Cas technology has markedly advanced research on organoids in terms of disease modelling and drug discovery. Isogenic disease models that mimic the disease profile observed clinically are created by introducing targeted mutations in the organoids, demonstrating the mechanism of genetic mutations and their influence on disease progression and treatment responses. For instance, CRISPR-Cas9 gene editing was applied to create malignant organoid tissues by introducing cancer-related mutations, essentially showing tumour initiation and development (Sekine, 2021).

Additionally, organoids offer an excellent platform for assessing the safety and effectiveness of gene corrections by CRISPR-Cas9. This method is particularly crucial for inherited diseases where precise mutation correction restores the normal function of the mutated gene (Geurts and Clevers, 2023).

Aside from direct gene editing, epigenome editing allows the creation of the epigenetic dysregulations observed in many diseases, including cancer, neurological disorders, and cardiovascular diseases, through precise modification of the epigenetic state of particular genes or genomic loci. These epigenetic manipulations are essential in disease modelling (Fadul et al., 2023). For instance, a CRISPR-Cas9-based demethylating platform, composed of a dCas9 fused with the TET1 catalytic domain, has been developed in cancer research and used to remove methyl groups from certain promoter regions. This technique restores gene expression and provides insights into epigenetic regulation in cancer. In a study, this CRISPR-based demethylation was applied to revert the epigenetic silencing of tumour suppressor genes like BRCA1, frequently linked to cancer progression (Choudhury et al., 2016).

Researchers have employed the CRISPR method to explore the regulatory function of intronic loci. For example, CRISPRa was used to activate the upstream intronic locus near the GCKR gene, and its involvement in type 2 diabetes (T2D) was confirmed. This study emphasises how genetic variations affect gene activity and add to the vulnerability of T2D (López Rodríguez et al., 2017).

Advancement in CRISPR-based drug discovery through gene-knockout studies, CRISPR high-throughput screening, and validation of drug efficacy in model organisms and organoids has greatly advanced our understanding of identifying new drug targets and determining complex relationships between genes and pathways. It has also paved the way for developing targeted and personalised therapies.

### 4.6 Regenerative medicine and tissue engineering

Stem cells, particularly pluripotent stem cells (PSCs), hold immense potential in regenerative medicine due to their ability to differentiate into various cell types. However, the therapeutic application of stem cells has been hindered by challenges related to their genetic stability, differentiation efficiency, and potential immunogenicity (Tabar and Studer, 2014). CRISPR-Cas technology addresses these issues by enabling precise genetic modifications that can enhance the therapeutic properties of stem cells (Zhang et al., 2017).

One notable example of CRISPR-Cas9’s application in regenerative medicine is its use to correct gene mutations in induced pluripotent stem cells (iPSCs) for treating genetic disorders. In a landmark study, CRISPR-Cas9 was employed to correct the duchenne muscular dystrophy (DMD) mutation in patient-derived iPSCs (Li et al., 2014). These corrected iPSCs were then differentiated into myogenic progenitor cells (MPCs) with restored dystrophin expression. These MPCs were transplanted into the MDM mouse model, expressing dystrophin in mouse muscles. The successful correction of the mutation and the subsequent differentiation into functional muscle cells exemplify the potential of CRISPR-Cas9 in regenerative medicine, offering a therapeutic strategy to treat DMD and other monogenic disorders (Jin et al., 2020). This approach has been demonstrated in generating cardiomyocytes from human embryonic stem cells (hESCs) by targeting and modifying key transcription factors involved in cardiac differentiation (Gähwiler et al., 2021).

Moreover, CRISPR-Cas allows for generating stem cell lines with enhanced resistance to immune rejection, a significant hurdle in stem cell transplantation. In a study, iPSCs from mice and humans were modified to reduce immunogenicity by inactivating MHC class I and II genes and overexpressing CD47. These hypoimmunogenic iPSCs retained pluripotency and differentiation ability, enabling them to generate endothelial cells, smooth muscle cells, and cardiomyocytes that evade immune rejection. Importantly, these cells survived long-term in fully mismatched allogeneic recipients without immunosuppression, suggesting the potential for universal, immune-compatible cell grafts (Deuse et al., 2019).

Tissue engineering aims to develop functional tissue constructs that replace damaged or diseased tissues. CRISPR-Cas technology enhances tissue regeneration by enabling the modification of critical genetic pathways that regulate cellular processes such as proliferation, differentiation, and ECM production. One of the critical applications of CRISPR-Cas in tissue regeneration is the enhancement of the regenerative capacity of endogenous cells. A pivotal study highlighted that CRISPR-Cas9-mediated activation of the Wnt/β-catenin pathway significantly improved muscle regeneration in animal models of muscle injury. This was achieved by targeting specific elements within the pathway to increase β-catenin levels in muscle stem cells, thus promoting their self-renewal and differentiation capabilities. The enhanced activation of this pathway led to an increase in the number of progenitor cells crucial for muscle fibre repair and regeneration (Mouradian et al., 2024). Moreover, CRISPR-Cas technology is promising for engineering cells to produce therapeutic factors in response to tissue injury. This approach could enhance regenerative medicine by enabling precise control over gene expression, allowing cells to generate necessary factors such as growth factors or cytokines only for tissue repair and regeneration (Hsu et al., 2019).

The application of CRISPR-Cas technology in regenerative medicine and tissue engineering represents a significant leap forward in developing therapies for various diseases and tissue injuries. Through precise stem cell modification and the enhancement of tissue regeneration, CRISPR-Cas not only addresses existing challenges but also opens new avenues for innovation in these fields.

### 4.7 CRISPR-Cas technology in medical diagnostics

One of the breakthroughs in CRISPR-Cas-based diagnostics is the development of CRISPR-based detection platforms such as SHERLOCK (Specific High-sensitivity Enzymatic Reporter unLOCKing) and DETECTR (DNA Endonuclease Targeted CRISPR Trans Reporter) (Chen et al., 2018; Kellner et al., 2019).

Both technologies can identify either RNA or DNA targets. Upon binding to its target RNA, Cas13 undergoes a conformational change and activates its non-specific nucleic acid cleavage activity, degrading the nearby RNA or DNA. When harnessed with reporter probes coupled with quenchers, this property produces a detectable signal, allowing for the sensitive detection of low-abundance nucleic acids (Kellner et al., 2019). In contrast, DETECTR uses Cas12 to target DNA. Like Cas13, Cas12 becomes active with its collateral cleavage activity by associating with its target DNA to cause cleavage in reporter probes adjacent to the target to generate a detectable signal (Chen et al., 2018). Both platforms harness the unique collateral cleavage activities associated with Cas12 and Cas13 for the amplification of detection signals, enabling precise and rapid identification of minute quantities of nucleic acids, rendering them priceless for several diagnostics applications ranging from viral detection to genetic mutation analysis (Bhardwaj et al., 2022).

SHERLOCK has been implemented to detect RNA viruses, including Zika and Dengue, and to identify bacterial pathogens, such as *Mycobacterium tuberculosis*. This platform combines CRISPR-Cas13a with isothermal amplification technologies, including recombinase polymerase amplification (RPA), facilitating rapid diagnostics and requiring simple laboratory infrastructure (Zahra et al., 2023). Likewise, DETECTR, using Cas12a, has found utility for detecting DNA viruses, including Human Papillomavirus, with excellent specificity and sensitivity (Yin et al., 2021).

CRISPR-based diagnostics are important platforms for applications in point-of-care testing, which is critical for managing outbreaks and providing timely medical intervention. More importantly, the portability, ease of use, and rapid turnaround time offer significant advantages over traditional PCR-based methods, particularly in resource-poor settings (Kaminski et al., 2021). Furthermore, the ongoing improvement of CRISPR systems for recognising a broader range of targets and integration with other diagnostic technologies, such as lateral flow assays and microfluidics, should increase their versatility. Medical diagnostics are greatly evolving with the advent of CRISPR-Cas technology, offering more sensitive, specific, and rapid approaches.

## 5 The status of CRISPR therapeutics in clinical trials

CRISPR-based therapeutics have evolved rapidly, with several candidates progressing through clinical trials and some nearing regulatory approval. These therapies are being developed to target a range of genetic and complex diseases through precise gene editing, which aims to correct underlying pathogenic mutations at the genomic level.

One of the most advanced CRISPR therapeutics is Casgevy (CTX-001) for the treatment of transfusion-dependent thalassemia (TDT) and sickle cell disease (SCD). CTX-001 is composed of edited hematopoietic stem cells (HSCs) using CRISPR-Cas9 to activate the fetal haemoglobin (HbF) gene, effectively compensating for defective adult haemoglobin (Sheridan, 2024). Recent clinical trial results have shown that patients treated with CTX-001 achieved sustained increases in HbF levels, significantly reducing or eliminating the need for blood transfusions in TDT patients (Frangoul et al., 2020).

EDIT-101 represents a significant milestone as the first *in vivo* CRISPR-based therapy to enter clinical trials (NCT03872479). EDIT-101 is designed to treat inherited retinal degeneration caused by a specific genetic mutation in the CEP290 gene, known as the IVS26 variant responsible for Leber Congenital Amaurosis type 10 (LCA10) (Maeder et al., 2019, p. 10; Leroy et al., 2021). This therapy utilizes an AAV5 vector, which has high tropism for photoreceptors, to deliver the gene-editing machinery directly to the retina. The vector carries DNA encoding the *Staphylococcus aureus* Cas9 (SaCas9) nuclease, along with two highly specific guide RNAs (gRNAs), and is driven by the GRK1 promoter, which is photoreceptor-specific. In a landmark study, this therapy is administered via a subretinal injection in patients with homozygous or compound heterozygous IVS26 variants, leading to targeted gene editing in photoreceptor cells. In the Phase 1-2 BRILLIANCE study, EDIT-101 was tested in 12 adults (ages 17–63) and 2 children (ages 9–14), with varying doses of the therapy. The primary goal was to assess safety, and secondary efficacy outcomes included changes in visual acuity, retinal sensitivity, and mobility. The results showed promising safety and tolerability, with no serious adverse events. Notably, 64% of participants showed meaningful improvements in at least one key visual function, with several individuals also demonstrating improvements in mobility and quality of life measures. These findings suggest that EDIT-101 holds potential for *in vivo* gene editing to treat inherited retinal diseases, specifically those caused by the IVS26 variant of CEP290 (Pierce et al., 2024).

A significant advancement in T1D cell therapy is the development of combination approaches using gene-edited allogeneic, immune-evasive stem cells. A Phase I/II trial is underway for VCTX211 (ClinicalTrial NCT05565248). VCTX211 comprises gene-edited allogeneic pancreatic endoderm cells modified with CRISPR-Cas9 and a perforated device for cell delivery and protection. This therapy includes two gene knockouts (B2M and TXNIP) and four insertions (PD-L1, HLA-E, TNFAIP3, and MANF) to enhance cell function, reduce immune rejection, and protect oxidative and inflammatory stress (Karpov et al., 2023). The trial is expected to be completed in August 2025.

Transthyretin (*ATTR*) amyloidosis is a serious disease caused by misfolded TTR protein tissue buildup. NTLA-2001 is a CRISPR-Cas9-based therapy that reduces serum TTR levels by targeting and editing the TTR gene in the liver. Preclinical studies confirmed a durable TTR knockout after a single dose. In a Phase 1 trial with six patients receiving either 0.1 mg/kg or 0.3 mg/kg, NTLA-2001 was generally well-tolerated with mild side effects. By Day 28, TTR levels dropped by 52% at the lower dose and 87% at the higher dose, indicating promising, dose-dependent reductions (Gillmore et al., 2021).

The effectiveness and efficacy of CRISPR therapeutics have also been proved in infectious diseases. EBT-101 is a CRISPR-Cas9-based antiviral therapy designed to cure HIV-1 based on targeting multiple HIV-1 genomic sites, inducing DSBs that remove large parts of the integrated viral DNA. EBT-101 is currently in a Phase 1/2 clinical trial of aviremic HIV-1-infected adults on stable antiretroviral therapy (ART). The primary goals are to assess the safety and tolerability of EBT-101, with secondary objectives focused on reducing HIV-1 DNA levels and achieving viral remission without ART. Preclinical studies have shown promising results, with effective excision of HIV-1 DNA in animal models. The ongoing clinical trial represents the first human application of CRISPR-Cas9 for HIV, with initial data suggesting the therapy is well-tolerated. However, conclusive results are pending (Presti et al., 2024).

LBP-EC01 is a CRISPR-engineered bacteriophage therapy developed by Locus Biosciences to treat UTIs caused by *Escherichia coli* (*E. coli*), including antibiotic-resistant strains. It combines six genetically enhanced bacteriophages with CRISPR-Cas3 technology, which targets and degrades bacterial DNA, enhancing its effectiveness and reducing resistance. In the first part of the Phase 2 ELIMINATE trial, various dosing regimens of LBP-EC01 were tested alongside oral trimethoprim-sulfamethoxazole (TMP-SMX). The results showed that LBP-EC01 was well tolerated, with no serious adverse events. Pharmacokinetic data revealed consistent drug levels in both urine and blood. Importantly, *E. coli* levels in urine decreased rapidly, and by Day 10, all evaluable patients had symptom resolution (Kim et al., 2024). These promising results support further investigation, with the trial’s second controlled phase now underway.

These CRISPR therapeutics represent a significant step forward in precision medicine, with promising early data from clinical trials suggesting the potential to fundamentally alter the course of genetic and infectious diseases. As CRISPR-based therapies advance, their clinical safety and efficacy will be critical in determining their potential to revolutionise treatment strategies for complex diseases. While numerous clinical trials have been conducted, detailed results are available in Supplementary Table 1, with only selected examples highlighted here due to the breadth of ongoing studies.

### 5.1 Data retrieval and processing

In August 2024, a systematic search for clinical trials related to CRISPR therapeutics was conducted using ClinicalTrials.gov, the International Clinical Trials Registry Platform (ICTRP), and the International Standard Randomised Controlled Trial Number (ISRCTN) databases (ClinicalTrials.gov, 2024; ISRCTN Registry, 2024; World Health Organization, 2024).

The search utilised both “CRISPR” as an abbreviation and the full term “Clustered Regularly Interspaced Short Palindrome Repeats” to capture all relevant studies. The data extraction focused on the key parameters: Study Numbers, Study Status, Conditions (general), Conditions (specific), Interventions, Phases, Start Date, and Completion Date.

The data was initially downloaded as separate datasets from the databases, combined into a single dataset, thoroughly filtered, and regulated. Finally, the duplicate registered trials were eliminated. Ultimately, 114 registered clinical trials were identified. The following section will give details and examples regarding some clinical trials categorised by disease types. Due to space limitations, elaborate discussions of all the trials are not given in the paper; however, they are described elsewhere by Zhang et al. (2023c).

### 5.2 Findings

The data analysis showed a broad distribution of CRISPR-based clinical trials across various disease categories (Supplementary Table 1). Cancerous diseases constitute 39.5% of the clinical trials, which is indicative of significant interest in the application of CRISPR-Cas technologies for the treatment of malignancies, such as leukaemia, multiple myeloma, and lymphoma, as well as gastrointestinal, lung, renal, and ovarian cancer, and sarcoma.

Blood disorders account for 28.9% of the trials, highlighting the application of gene editing in conditions like thalassemia and sickle cell disease, where CRISPR-Cas offers potential curative therapies. For instance, therapies like EDIT-301 and CTX001 aim to correct genetic mutations in hematopoietic stem cells to produce functional haemoglobin (Christakopoulos et al., 2023; Hanna et al., 2023). Eye diseases comprise 3.5% of the trials, focusing on genetic conditions affecting vision such as primary open-angle glaucoma, neovascular age-related macular degeneration (nAMD), retinitis pigmentosa, and Leber congenital amaurosis 10.

Genetic disorders affecting multiple organs constitute 4.4% of the trials, addressing a variety of inherited conditions, including hereditary angioedema, transthyretin-related familial amyloid polyneuropathy, transthyretin-related familial amyloid cardiomyopathy, wild-type transthyretin cardiac amyloidosis, Kabuki syndrome 1, Rubinstein-Taybi syndrome, and pyruvate kinase deficiency. Infectious diseases represent 6.1% of the trials, focusing on CRISPR-Cas applications for pathogens such as HIV-1, viral keratitis, HSV, carbapenem-resistant Enterobacteriaceae, COVID-19, and gastrointestinal infections. Metabolic disorders, including diabetes mellitus (type 1), elevated lipoprotein, and refractory dyslipidemias, comprise 3.5% of the trials. Other conditions comprise 6.1% of the trials, encompassing various diseases.

**FIGURE 3:**
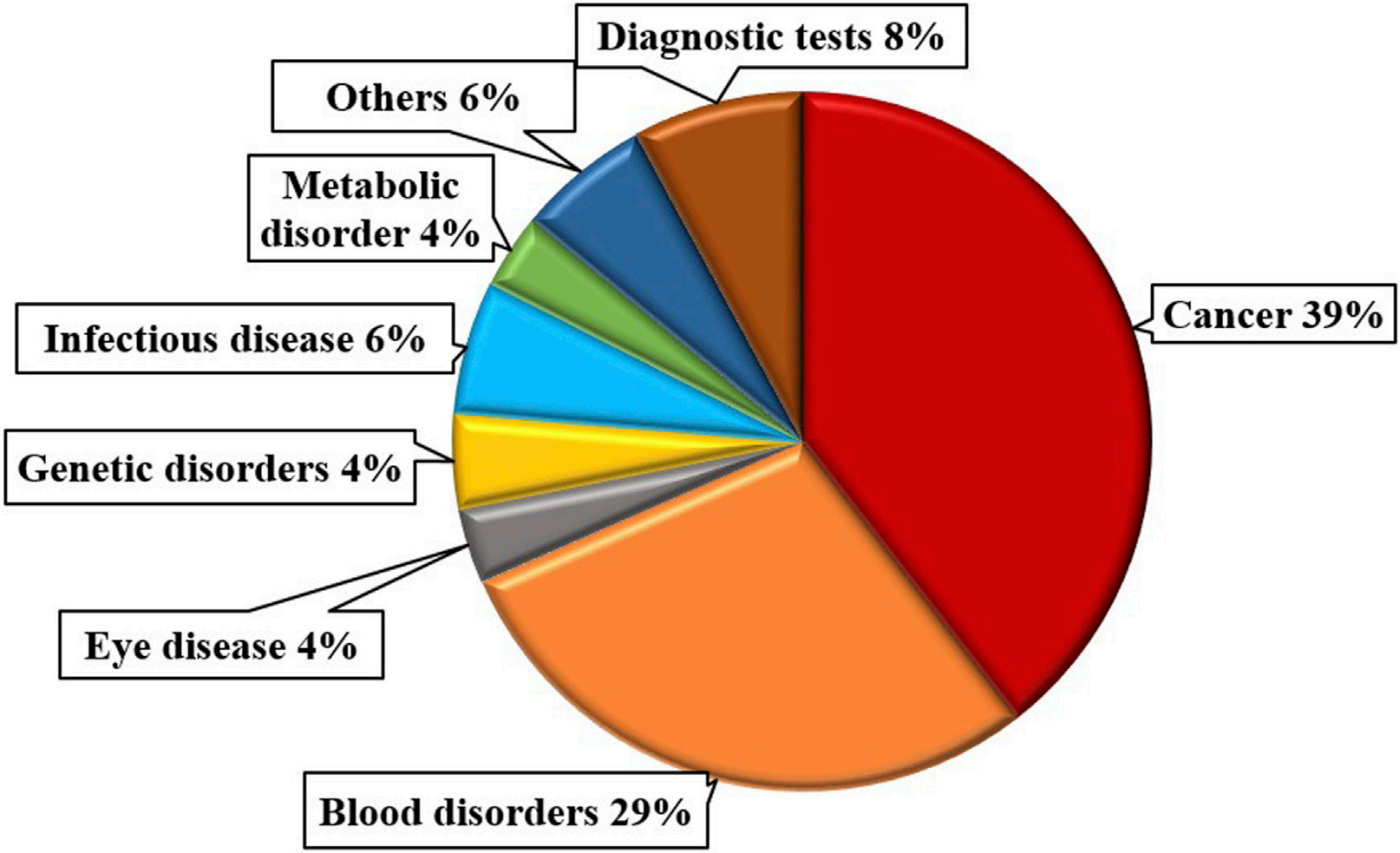
Distribution of CRISPR-Cas clinical trials by therapeutic area.

Finally, diagnostic tests account for 7.9% of the trials, emphasising the role of CRISPR-Cas in developing new tests for accurate and rapid disease detection, including tuberculosis, pulmonary infections, enterovirus infections, pertussis, asymptomatic COVID-19, SARS, and aspergillosis. The results of this comprehensive search are illustrated in Figure 3.

Most trials are categorised under Phase 1, with 33 focusing primarily on early-phase safety and dosage assessments. A notable number of trials, 21, span both Phase 1 and Phase 2, indicating a combined evaluation of initial safety and preliminary efficacy. Fewer trials bridge Phase 2 and Phase 3, with only two trials covering both exploratory and confirmatory phases. Trials solely in Phase 3 are less common, with five studies dedicated to large-scale confirmation of efficacy and safety. Lastly, 36 clinical trials show no clear information in the databases.

These results indicate a significant focus on cancer and blood disorders; however, they are still in the early phase of the trials. Clinical trials also show that CRISPR-Cas technology in medical diagnostics is also evolving, especially in the diagnosis of viruses. Nevertheless, the gaps in phase reporting and many trials lacking clear information underline the required improved standardisation and transparency to fully realise CRISPR-Cas’s therapeutic potential.

## 6 Challenges, safety issues and ethical concerns

One of the primary concerns involves the potential for off-target effects, where unintended genomic alterations occur, leading to unpredictable and possibly harmful consequences. The off-target risk represents a prominent challenge despite the significant improvements in specificity and precision of CRISPR-Cas systems. Often, inadvertent changes in genomic loci disrupt normal gene function or activate oncogenes, leading to entirely new diseases or exacerbating the existing conditions (AYANOĞLU et al., 2020). Another challenge is the efficient delivery of CRISPR-Cas components into the target cells or tissues. The delivery system must ensure the safe delivery of CRISPR-Cas machinery into the intended cells without degradation or unwanted immunological reactions. Different delivery systems are being explored, including viral vectors, such as adenoviruses and lentiviruses, and non-viral methods, such as lipid nanoparticles. However, each method has its limitations. Viral vectors, though efficient, can integrate into the host genome and potentially cause detrimental genomic mutations. Nonviral techniques, on the other hand, often lead to low delivery efficiency and poor targeting specificity (Sioson et al., 2021). Furthermore, CRISPR-Cas components can potentially provoke an immune response in the patient. Since Cas proteins, particularly Cas9, are derived from bacteria, the human immune system may recognise them as foreign and mount an immune response. This could lead to inflammation or rejection of the treated cells (Rasul et al., 2022).

The ethical concerns surrounding CRISPR-Cas technology are profound and multifaceted, reflecting the transformative potential of this gene-editing tool. Germline editing of human embryos presents significant ethical concerns, primarily regarding its long-term impact on future generations. While it could eradicate certain genetic disorders, it also risks introducing new abnormalities and altering human evolution unpredictably. This raises ethical issues, including potential inequalities where only some could benefit from these advances (Lanphier et al., 2015). Informed consent is a fundamental ethical requirement, yet it is challenging in the context of CRISPR-Cas therapies. Patients must be thoroughly informed about the risks and benefits, a task complicated by the technology’s rapid development and uncertain long-term effects (Gonzalez-Avila et al., 2021). Moreover, equitable access to CRISPR-Cas treatments is also a major concern. The high cost of these therapies could restrict their availability to wealthy individuals or nations, worsening health disparities. Ensuring broad access is essential to avoid exacerbating inequalities (Lorenzo et al., 2022).

Finally, using CRISPR-Cas for non-therapeutic enhancements, such as boosting intelligence or physical abilities, raises ethical questions about genetic intervention limits and societal implications. Careful regulation is needed to address fairness and redefine “normal” versus “enhanced” human traits (Gabel and Moreno, 2019).

While CRISPR-Cas technology holds promise for correcting genetic disorders, it is vital to address these ethical concerns—off-target effects, germline editing implications, informed consent, access equity, and potential misuse for enhancements—to use the technology responsibly. Several promising approaches are being explored to address current challenges and improve the efficacy of CRISPR-Cas systems in clinical applications. Ongoing research aims to enhance the precision of CRISPR-Cas systems by developing next-generation Cas proteins. Engineered variants such as Cas9-HF1 and eSpCas9 have demonstrated reduced off-target activity, improving overall specificity (Allemailem et al., 2023).

Future advancements in delivery technologies will be crucial for the clinical success of CRISPR-Cas therapies. Improved non-viral methods, such as tissue-targeted nanoparticles, promise safer and more effective delivery. Additionally, new viral vectors with enhanced safety profiles and reduced risk of insertional mutagenesis are under exploration (Lu et al., 2023).

Less immunogenic Cas variants and humanised Cas proteins are being developed to address immune responses. Transient delivery methods, where CRISPR components are quickly degraded, may also minimise immune reactions (Ewaisha and Anderson, 2023). Responsible use of gene-editing technologies is essential, particularly to mitigate off-target effects and ensure long-term safety. Clear regulatory guidelines and oversight will be necessary to prioritise patient safety and ethical considerations (Gonzalez-Avila et al., 2021). While significant challenges remain, the ongoing advancements in CRISPR-Cas technology, including improvements in specificity, delivery, and immunogenicity management, pave the way for its broader application in treating a wide range of diseases. The combination of technical innovations and thoughtful, ethical oversight holds promise for the future of precision medicine, where CRISPR-Cas could become a cornerstone in treating genetic disorders, cancers, and other diseases.

## 7 Conclusion

The current study highlights the transformative impact of CRISPR-Cas technology on modern medicine, particularly in precision medicine, therapeutic development, and genetic disease correction. Advancements in CRISPR-Cas systems, including high-fidelity Cas9, prime and base editors, and Cas12 and Cas13, have revolutionised genome editing. These innovations enable precise modifications that were previously unattainable. Such advancements have been pivotal in drug-target discovery, oncology, regenerative medicine, and infectious diseases. Developing the new class of epigenome editors allows treatment without direct editing of DNA. It holds great potential, while preclinical studies have been efficient in this approach, which is safer, more reversible, and easy to adapt to multiple conditions. The study underlines the potential of CRISPR-Cas technology in altering inherited genetic disorders, shedding new light on untreatable conditions. Successful application in clinical trials, including the FDA approval of Casgevy for sickle cell disease, highlights its promise in therapeutic interventions. However, with these new developments, there are challenges such as off-target effects, ethical issues, and refinement in delivery mechanisms. It may be important to resolve these challenges for future research to help unlock the potential of CRISPR-Cas technology in a clinical scenario, resulting in more personalised, effective, and safe medical treatments.
